# Experiences of becoming widowed in old age – a cross-countries study with qualitative interviews from Denmark and quantitative measures of association in a Swedish sample

**DOI:** 10.1080/17482631.2020.1871181

**Published:** 2021-02-04

**Authors:** Christina Blanner, Anja Elliott, Peter Hjorth, Jens Søndergaard, Cecilia Mattisson, Kjeld Andersen

**Affiliations:** a Department of Mental Health Odense, Mental Health Services in the Region of Southern Denmark, Odense, Denmark; b Department of Clinical Research, University of Southern Denmark, Odense, Denmark; c Department of Psychiatry, Mental Health Services in the Region of Southern Denmark, Esbjerg, Denmark; d Department of Regional Health Research, University of Southern Denmark, Odense, Denmark; e Centre for Psychiatric Nursing and Health Care Research, Odense, Denmark; f Research Unit for General Practice, Department of Public Health, University of Southern Denmark, Odense, Denmark; g Division of Psychiatry, Department of Clinical Sciences Lund, Faculty of Medicine, Lund University, Lund, Sweden

**Keywords:** Bereavement, cohort study, experience, mental health, mixed-methods, multi-methods, old people, qualitative description, Scandinavia, widowhood

## Abstract

**Purpose**: Becoming widowed is a stressful health-threatening event causing major life changes. We explored how widowed people experience becoming widowed and examined if these experiences are quantitatively associated with widowhood.

**Methods**: A multi-methods study using an exploratory sequential mixed-methods approach including a qualitative descriptive study with widowed people from Denmark and a Swedish cohort study. Qualitative interviews (n = 9) were analysed using qualitative content analysis, describing experiences as explained by the widowed people. The quantitative association of the experiences was examined by identifying proxies for the qualitative experiences of widowhood in the cohort study and examining the occurrence in widowed people compared to married people (n = 1,095).

**Results**: Six categories of experiences emerged: the circumstances around spousal death, mental health and well-being, physical health, social relations, activities and practicalities. The quantitative examination showed a significant association with widowhood regarding mental and physical health problems.

**Conclusion**: The circumstances around spousal death and the time before spousal death, in general, were important to how participants felt being widowed. Being ill negatively affected mental health and well-being, partly because of the inability to participate in activities and social relations. This is important, as health problems are more common among widowed people than married people.

## Introduction

Losing a spouse to death is a common life event especially in old age (The Loomba Foundation, 2015). It is associated with grief—an emotional response which is normal and natural in relation to loss and major life alterations (Hardy-Bougere, 2008). Nonetheless, becoming widowed is known to be one of the most painful and stressful life events, causing high levels of distress (Stroebe et al., 2017; Shear, 2015).

The normal process of grief is sometimes described as a five-stage process (Maciejewski et al., 2007), however, it is important to consider, that the spectrum for normal emotional response is wide, and varies on both a cultural as well as individual level (Hardy-Bougere, 2008; Zisook & Shear 2009). Whereas for most people the distressing symptoms of grief decrease over time, some people experience persisting intense pain and yearning for the loved one, and the normal emotional response becomes disabling, resulting in prolonged or complicated grief disorder (Shear, 2015). Besides the emotional distress in terms of grief, losing a spouse is also associated with adverse effects on the health including increased risk of acquiring chronic and acute diseases (Stroebe et al., 2017), such as cardiovascular diseases (Einiö & Martikainen, 2019), diabetes and arthritis (Van den Berg et al., 2011). Furthermore, mental health problems including depression and anxiety disorders are known to be common in widowed people (Kristiansen et al., 2019a, Kristiansen et al., 2019b).Ultimately, widowhood is associated with increased mortality (Moon et al., 2011; Shor et al., 2012).

Although old age is generally associated with functional and sensory decline and an increasing occurrence of health problems (Jaul & Barron, 2017), the adverse effects on health and even mortality in widowhood cannot be attributed to the natural course of ageing, as these problems are evident also when comparing widowed people to people who are married of the same age (Blanner et al., 2020; Einiö & Martikainen, 2019; Valdimarsdottir et al., 2003; Wilcox et al., 2003).

Though widowhood is associated with negative outcomes of health, it is important to consider that widowhood is not a disease itself (Stroebe et al., 2017) and neither is grieving (Hardy-Bougere, 2008; Shear, 2015), and the uncomplicated process of grief does not require treatment or professional interventions (Zisook & Shear 2009). In fact, intervening especially in the early stages of the normal process of grief can be damaging (Zisook & Shear, 2009). Instead, preventive interventions to improve mental health and well-being in widowhood must be designed specifically to those who are most at risk of developing adverse outcomes in relation to widowhood (Nseir & Larkey, 2013; Schut & Stroebe, 2005). In order to do so, we need to understand possible pathways into the adverse outcomes of widowhood and identify who is at special risk of developing these.

Previous research has examined possible risk and protective factors regarding the adverse effects on health and their association with widowhood (Yaolin. et al., 2019) including coping strategies (Carr, 2018; O’Rourke, 2004), financial strain (DiGiacomo et al., 2015) and the influence of social relations (Anusic & Lucas, 2014; De Vries et al., 2014) such as support from family members and co-residence with children (Jeon et al., 2013; Kyung Do & Malhotra, 2012; Zhou & Hearst, 2016). However, little is known on how these factors are associated with each other, and how they interact with the outcomes of widowhood (Stroebe et al., 2017). As widowhood alters several aspects in life simultaneously (Nseir & Larkey, 2013), studies on association analyses in widowhood are an important way to increase this understanding. Therefore, we conducted a qualitative descriptive study combined with a quantitative examination exploring experiences of widowhood and their possible associations with each other, and how these are occurring in widowed people compared to married people.

The aims of the study were to 1) explore qualitatively how becoming widowed is experienced by the widowed person, 2) examine if the identified experiences of widowhood are quantitatively associated with being widowed compared to being married.

## Material and methods

### Design

The study was a multi-method study conducted in two parts using an exploratory sequential mixed methods approach as described by Kettles et al. (2011). First, a qualitative descriptive study exploring experiences of widowhood was conducted. Subsequently, a longitudinal cohort study, based on a different sample, was used to quantitatively assess how the experiences described in the qualitative interviews occurred in widowed people compared to married people.

The combination of the two studies was enabled by identifying questions in the Lundby questionnaire that served as quantitative proxies for the experiences of widowhood expressed by the participants in the interviews.

Furthermore, the study used a cross-countries design using data from both Denmark and Sweden. This combination is meaningful, as the two countries are similar sociocultural and regarding the structure of the welfare system (Daatland, 1994; Iqbal & Todi, 2015). Combining and comparing data from the two countries is therefore common practice in health research. The mixed methods design integrating the findings from Danish qualitative interviews, with pre-existing Swedish quantitative data enables us to address the transferability of the findings from the qualitative interviews, thus strengthening the trustworthiness of the study (Elo et al., 2014).

### Ethics

The entire study, including both the qualitative and quantitative part, is approved by the Danish Data Protection Agency in the Region of Southern Denmark (Reference: 2008–58-0035, Journal number 16/31735). Interview studies do not require formal approval from the National Committe on Health Research Ethics (2019).

### The qualitative part of the study

#### Setting

The qualitative descriptive study was conducted in Denmark. Denmark is a Scandinavian country with a total population of 5.8 million people (Statistics Denmark—Statsbank, 2019). Denmark has a strong national welfare system, with universal access to health care, free from of up-front charge as it is paid over taxes (Pedersen et al., 2012). This includes access to general practitioners as well as more specialized treatment from office-based specialists and hospitals (Pedersen et al., 2012). Furthermore, services such as help with cleaning and personal care, home nursing and rehabilitation are provided free of charge via the municipalities for people who are referred to these services by a social worker. The municipalities furthermore offer services as transportation of citizens and delivery of precooked meals for people in the need for these services. These are not free of charge but must be paid for by the citizen; however, there are national fixed maximum prices, and these are non-profit services. In general, the services available are equal across municipalities as well is the health care provided.

Most participants in the interviews were from Odense municipality and were living either in this city or in the suburbs (n = 5). Odense is the third largest city in Denmark, with about 180,000 citizens (Statistics Denmark—Statsbank, 2019). The remaining participants lived in minor cities in Jutland (n = 2) or Funen (n = 2), all with facilities such as grocery shopping, a local senior citizen house, and a church in the nearby community.

#### Participants

Participants were sampled purposeful by recruiting participants from different settings in the healthcare system and the community to increase the likelihood of people having different experiences of widowhood (Bradshaw et al., 2017; Sandelowski, 2000). Recruitment was conducted by general practitioners who met eligible participants in their consultation. The general practitioners acted only as facilitators of the contact between the participant and the interviewer and had no further involvement in the study. Furthermore, participants were recruited from the psychiatric facility where the primary and senior investigator were employed. The interviewer (the primary investigator) did not have any knowledge of or relationship with any of the participants prior to the interviews and did not engage in such afterwards. Finally, as public knowledge dissemination, the primary investigator gave public lectures about widowhood at the local library and similar places. The qualitative interview study was mentioned at the end of these presentations, and interested people were invited to participate.

The criteria for participation were: participants had to be about 60 years of age or older, speak Danish, and have been widowed for less than two years. The latter to ensure proximity to the experience of becoming widowed. There were no predefined exclusion criteria, and no participants referred to the interviewer were deemed ineligible to participate.

#### Interviews

All interviews were conducted by the first author (CB), who had no prior relation to any of the participants. All interviews except one were conducted in the participants’ homes. One interview was conducted at the interviewers’ research office, as this was preferred by the participant. The interviews were conducted as unstructured interviews. That is, there was no interview-guide prepared prior to the interviews, except for the opening statement, which was the same in every interview: (translated from Danish): “What I would like to hear about, is how it has been for you since your spouse passed away”. After this statement, the interviewer paused and let the participants talk. This method was chosen to gain the richest descriptions from the participants’ own perspectives (Kvale & Brinkmann, 2009). During the interviews, the interviewer asked for meaning clarifications, posed supplementary questions and redirected with structuring questions if the participant went far from the topic (Kvale & Brinkmann, 2009), but generally followed the story the participants decided to tell. As recommended by Elo et al. (2014) conducting interviews and analysis were partly iterative. After the first five interviews were conducted, two investigators made a preliminary discussion of themes in the interviews. During the remaining interviews, the interviewer asked elaborately about these themes if they were mentioned by the participants or could be introduced as a natural part of the conversation. Interviews were conducted until no new information seemed to emerge from conducting more interviews (Bradshaw et al., 2017). The interviews were audio recorded and transcribed verbatim by the first author. The accuracy of the transcripts was checked by another investigator (AE) by listening to the audio recordings and any discrepancies were corrected (Kvale & Brinkmann, 2009).

#### Analysis

The analysis was conducted according to the inductive qualitative content analysis as described in the guidelines by Elo and Kyngäs (2008).

Three investigators (CB, AE & PH) participated in the analysis. The analysis consisted of five steps. Table I shows the flow of the analysis with concrete examples from the analysis. Step 1) “Open coding”: Each investigator independently read the interviews. The investigator marked meaning units and assigned the meaning unit with a preliminary code, describing the content of the meaning unit. A meaning unit could be a longer passage or a sentence. Only manifest content was included to minimize interpretation. That is, only spoken words were used in the meaning units and laughing, crying or breaks, etc. were not considered (Elo et al., 2014). The preliminary codes used were defined by the investigator while reading the interviews. Each investigator reread each interview, until no new meaning units were found. Step 2) Each interview was assessed by the three investigators in a discussion. Each marked meaning unit and the preliminary code describing the content of the meaning unit were discussed, to ensure agreement upon the meaning of the content between the three investigators. It was decided prior to the analysis, that if disagreement would occur, a fourth investigator would be asked; however, in no cases, did the three investigators not agree upon the meaning of meaning units or use of certain preliminary codes describing them. Step 3) The preliminary codes were assessed in a discussion by all three investigators, merging and condensing the preliminary codes into new codes. This was done until no further meaningful merging was possible. 4) The content and meaning of the codes were discussed and codes were organized into categories. The categories were defined as an overall description of the content of the codes and were defined during discussion by the three investigators. 5) The primary investigator drafted the description of the categories, and this was revised until agreement by the two other investigators conducting the analysis.

**Table I.** Flow of the qualitative analysis with description of each step and concrete examples from the analysis

Steps of analysis	Description of example	Example from the analysis
**Step 1**: Individual reading of interviews, conducting open coding: marking of meaning units and defining preliminary codes.	Example showing a piece of a transcribed interview with a marked meaning unit (underlined). The meaning unit was assigned the preliminary code “new tasks and responsibilities” by the investigator.	…sometimes you need to remind each other something “what am I going to wear to that funeral?”, you know? Both parts can help each other.. These small trivial things.. You are totally responsible for everything, whereas previously you literately had a division of the duties and responsibilities. That is hard to get used to….
**Step 2**: Assessment of interviews, reviewing meaning units and preliminary codes by the three investigators	–	–
**Step 3**: Merging and condensation of preliminary codes into codes defined during discussion by the investigators	Example showing some of the preliminary codes which were defined individually by the investigators during step 1, which were condensed into the code “Loneliness” during discussion by all three investigators.	“the house is empty” “doing things alone” “the silence”
**Step 4**: Organizing codes into categories	Example showing the codes which were defined by the three investigators during step 3, which were organized into the category “mental health and well-being”.	“Anxiety” “Loneliness” “Speculations” “Feeling tired” “Difficulties sleeping” “Suicidal thoughts” “Stress” “Grief”
**Step 5**: Written description of categories by the primary investigator, revised by the two other investigators until agreement	–	–

Examples from the interviews are translated from Danish to English. To ensure confidentiality examples from the interviews are kept as short as possible to avoid reporting too many and too long passages from a single interview. It was not possible to exemplify step 2, as this was a step of discussion between the three investigators. Similarly, there is no example of step 5 as the outcome of step 5 is the written presentation of the qualitative findings as presented in the paper.

Although discussion of the meaning of the content was necessary during the analysis to ensure all investigators had the same understanding of the content, a strictly descriptive focus was maintained in the analysis, keeping interpretations to a possible minimum and referring to actual phrases used by participants (Chafe, 2017), thereby ensuring the conformability of the findings (Elo et al., 2014).

#### Considerations of trustworthiness

The primary investigator was a medical doctor (MD) and PhD student working within the field of psychiatry. The primary investigator had concurrent with the present study examined common mental disorders (Kristiansen et al., 2019a, 2019b) and mortality in widowhood (Blanner et al., 2020) and thus had a pre-existing understanding of adverse effects of widowhood. The primary investigator had experience in conducting qualitative research from a previous study (Blanner Kristiansen et al., 2015). PH (associate professor) had participated in the aforementioned studies of common mental disorders in widowhood (Kristiansen et al., 2019a, 2019b) and was experienced in qualitative research (Juel et al., 2018; Blanner Kristiansen et al., 2015; Weiser et al., 2009). AE (MD) did not have academic experiences with widowhood and was not experienced in conducting qualitative research. This group of investigators for the qualitative analysis was chosen to ensure different backgrounds and preunderstandings of the phenomenon.

A “practice session” was held to ensure that the method of analysis was consistent by the three investigators as recommended by Elo et al. (2014) to ensure the credibility of the analysis. This was conducted after all the interviews were conducted. The practice session consisted of a small part of one of the interviews, where the investigators performed step 1 and 2 of the analysis. The purpose was discussing any difficulties or unclarities in the method of analysis, rather than discussing the content of the interviews. Subsequently, the investigators performed the analysis according to the steps above on all interviews, including also a reassessment of the small part which had been used in the practice session.

Presentation of data was revised by two investigators referring to the original data material to ensure that the final presentation remained descriptive and close to the actual words used by the participants (Bradshaw et al., 2017; Neergaard et al., 2009), ensuring conformability of the findings (Elo et al., 2014).

#### Ethical considerations

Several ethical considerations were taken to accommodate with the sensitive nature of the research area, which bereavement research is known to be, as well as the vulnerability of the participants being of older age (Butler et al., 2019). The general practitioners, who had referred participants, were not informed on whether or not their patient chose to participate, and the participants were informed of this prior to participation, ensuring that they did not feel pressured to participate to please their general practitioner. Participants had time for changing their mind from agreeing to participate and until the interview, and this was mentioned to them in the telephone when arranging the interviews. The participants gave informed consent and were informed that they could withdraw this at any time during and after the interview. Names and contact information were only used to arrange the interviews. The interviews were kept anonymous and all names and recognizable places were censored in the transcripts. As the interview could potentially evoke emotional distress, due to the sensitive nature of the topic discussed (Butler et al., 2019), it was ensured that all participants had a safety net after the interviews. Participants had the interviewer’s contact information and were encouraged to make contact, also outside working hours, if the interview had evoked any uncomfortable thoughts or emotions or if they felt they needed a follow-up talk. None of the participants made use of this opportunity.

### Combining the qualitative findings with the quantitative data

The study used a sequential approach to integrate the qualitative and quantitative data (Kettles et al., 2011). That is, after conducting the qualitative analysis, the Lundby study was used to identify quantitative proxies for the experiences described in the qualitative analysis. To ensure independency within the two studies, and to minimize the risk of interviewer bias during the qualitative interviews and during the qualitative analysis, the assessment of the quantitative data was not conducted until the qualitative analysis was completely finished. Thus, the quantitative data material did not influence on the qualitative part of the study.

### The quantitative part of the study

#### The Lundby study

The Lundby study is a longitudinal follow-up study over 50 years conducted in southern Sweden (Nettelbladt et al., 2005). Four waves have been conducted in 1947, 1957, 1972 and 1997, respectively. The original aim of the Lundby Study was to examine personal traits and morbidity in a general population by observing an entire population in a community (Henderson & Jablensky, 2010). The interviews were conducted by experienced psychiatrists and were based on face to face interviews with participants, use of key informants and data gathered from out-patient clinics, general practitioners and from 1997 also information about in-patient treatment from national registers (Henderson & Jablensky, 2010). The first wave conducted in 1947 included 2,550 people aged 0–92 years. The total Lundby study included 3,563 participants. New people were added to the cohort in 1957. In the 1972 and 1997 follow-up, only the people from 1947 and 1957 were followed, and no newcomers were added (Nettelbladt et al., 2005). Further details of the method in the Lundby study are described elsewhere (Henderson & Jablensky, 2010; Nettelbladt et al., 2005).

#### Sample

For identification of participants for the present study, the 1972 and 1997 follow-up were used. Participants registered as married in the 1972 follow-up were identified. Participants who were alive and still registered as married in the 1997 follow-up were included as married in the present study. Those who were married in 1972, and still alive but widowed in the 1997 follow-up were included as widowed.

#### Quantitative proxies for qualitative experiences

For the quantitative analysis data from the 1997 follow-up were used. We identified questions in the 1997 questionnaire (see Supplementary File 1) that were concerned with the same topic as the categories and codes generated in the analysis of the qualitative interviews. Thus, the questions identified in the Lundby study served as quantitative proxies for the experiences described by the widowed persons in the qualitative interviews.

#### Quantitative analysis

The quantitative proxies identified in the Lundby study were analysed according to marital status. This was done by conducting descriptive statistical comparisons in the sample identified in the Lundby study, comparing the distribution of the answers in married and widowed people. For continuous measures comparisons of the means were calculated using two samples t-test. Chi squared test was used for categorical measures. For ordered categories, chi squared test for trend was used. Comparative analyses by marital status were conducted by listwise deletion of missing data. All analyses were conducted in the statistical software Stata-16 (StataCorp, 2019). A significance level of p < 0.05 was used.

## Results

### Qualitative analysis

#### Characteristics of the participants

The study included nine qualitative interviews, of which six participants were widows and three participants were widowers. The participants were between 62 and 90 years old. All participants lived alone in their own homes (not institutionalized), which for all participants was the same home as where they had lived with the deceased spouse. The participants had been widowed between two months and two years at the time of the interview. The participants reported the following causes of death from the deceased spouse: dementia (n = 2), cancer (n = 4), liver failure (n = 1), sudden cardiac arrest or acute myocardial infarct (n = 2). Two participants described the death of the spouse as sudden and unexpected, whereas the remaining seven participants described that the death of the spouse was more or less expected due to long-term illness. The interviews were conducted between February 2017 and July 2018. The interviews lasted between 51 and 101 minutes.

#### Overall observations from the qualitative interviews

Six categories of importance in widowhood emerged (see Table II). The categories were: (I) The circumstances around spousal death (II) Mental health and well-being (III) Physical health (IV) Activities (V) Social relations and (VI) Practicalities.

**Table II.** Categories and related codes from the qualitative analysis

**I. Circumstances around spousal death**	**II. Mental Health and well-being**	**III. Physical Health**
*• Issues due to sudden death* *• Issues due to a long course of illness* *• Thoughts about regret and guilt*	*• Loneliness* *• Anxiety* *• Speculations* *• Feeling tired* *• Difficulties Sleeping* *• Suicidal thoughts* *• Stress* *• Grief*	*• Physical constraints* *• Lack of energy* *• Affects mental health and well-being*
**IV. Activities**	**V. Practicalities**	**VI. Social relations**
*• Facilitator of well-being* *• Activities in the community*	*• Practicalities concerning the death of the spouse* *• Responsibilities and duties*	*• Emotional support* *• Practical support*

An observation which emerged across all interviews and which was general for most of the experiences described by the participants was the influence of the time before spousal death. Although all participants were asked to tell how it had been for them since they lost their spouse, they generally spoke about both the time before and after the spousal death throughout the interviews and across different topics. This was especially true regarding the circumstances around the spousal death (see description below), however, not exclusively around this topic. For an example, it was also stressed that participating in activities was important not only after becoming a widow(er), but also during a long course of illness of a spouse, to maintain one’s own mental health and well-being. Similarly, also having been able to deal with practicalities, such as the will, perhaps selling of the house, etc. prior to spousal death was mentioned as important for the bereaved. These examples were mentioned as important, as they influenced on the mental health and well-being of the widow(er), and thus how one was able to cope with being widowed.

*(I) The circumstances around spousal death*

You can say, that the process actually begins towards the end where she is still alive but isn’t really present at this planet anymore. (participant 1)

This was the most prominent category, taking up most of the time in all interviews. All participants started out by talking about the circumstances around the spousal death and subsequently the time after death.

Most participants had experienced a course of illness of the spouse before his or her death. All the participants where the spouse have had a long course of illness explained that this was straining. Some mentioned that being a caregiver was a burden in several ways. On one hand, you were already alone because you had all the responsibilities around the household. On the other hand, you were tied, as the spouse depended on you day and night. Therefore, some mentioned that becoming widowed was a relief. Others did not mention the feeling of relief but emphasized that the course of illness before the death of the spouse had drained them from all energy. One widow said after describing a long course of illness: “*all that did that in the time of grief I had no energy*” *(participant 2)*. Besides not having the energy to cope with grief, some mentioned, that they had deliberately postponed dealing with their own health problems because they needed to be able to take care of their spouse: “*That is also why I postponed my surgery because I knew that if I had to undergo surgery, then I wouldn’t be able to drive a car” (participant 2).*

Some of the thoughts which occupied the participants were thoughts about regrets and guilt concerning their actions around the death of the spouse. One mentioned that this was tormenting her, and *“in the beginning it was both grief and the feeling, that I failed him. That was the worst. (participant 2)”* Some participants mentioned things they regretted not doing differently or having thoughts about whether or not things would have been otherwise, had they done so.

Some participants mentioned that they experienced poor professional support during the spouse’s illness. Three participants who had been working in health care felt that professional mistakes and humiliating approaches in the treatment of their spouse were overwhelming for them and degrading for their spouse. This made them feel unsafe and spend even more time caregiving.

On the contrary to a long course of illness, those who experienced sudden death of their spouse mentioned not having said goodbye as the worst thing. They did, however, also mention that they felt relief that the spouse did not suffer a long and painful course of illness.

*(II) Mental health and well-being*

I can tell you, as a widow it is pure survival everyday (participant 2).

The mental health and well-being of the participants was discussed in all of the interviews. Within this category, eight codes emerged representing different emotions and problems related to the participant’s mental health and well-being. The codes were loneliness, anxiety, speculations, feeling tired, difficulties sleeping, suicidal thoughts, stress, and grief.

Most participants expressed being widowed as difficult. Loneliness was mentioned as the worst thing being widowed: *“that is probably the loneliness. I’m still left with that, when I have been somewhere, out for a walk or something else, and step inside and close the door. Then, this total silence. Boo! You step inside, and there is completely silent and empty” (participant 4)*. This silence was mentioned by several participants. They emphasized the feeling that you did not have someone to talk to, and to experience things in life with. Some participants associated this with being lonely, whereas one participant said: *“I don’t feel that I am lonely, but I do miss that counterpart to talk to, and be with, and to tell things when I have experienced something” (participant 9)*. Thus, being alone and feeling lonely was experienced differently by the participants.

The silence and emptiness had made some of the widows more anxious than before the spouse had died, as they no longer felt safe when it was dark outside. This hindered one widow from participating in social events and activities, if it meant that she would be going home in the dark.

Some participants mentioned that participating in social events was especially difficult because people would often come as a couple, and you would feel like the odd one. Furthermore, the holidays and weekends were mentioned as especially difficult. This was both due to the fact that you would often feel even more alone, if you did not have someone to be with. It was exemplified that if you did not have any invitations then you would just be alone, whereas when you were a couple, you always had each other.

Being alone most of the time gave rise to more speculations than usual. Speculations about one’s identity and wishes for the future were mentioned. A few participants even said that suicidal thoughts had been present. One mentioned that she had had thoughts of suicide because it would be the easy choice: *“then I wouldn’t have to be alone with all the speculations about the house and stuff (.) you have to maintain it etc. and we used to be together in this. I would escape from that if I was not here” (participant 6).*

*(III) Physical Health*

It is difficult to keep your spirits up when you are not well, I think (participant 5).

It was mentioned, that physical health problems were an important factor of how one is able to manage widowhood. One mentioned that the practical responsibilities around the house felt even more burdensome, because she was not physically well, and therefore could not handle this.

Furthermore, participants mentioned poor health as an important barrier for engaging in social activities, both due to not having the energy to participate and due to physical constraints, such as not being able to drive a car, which prevented them from going places.

Some mentioned that poor physical health was associated with not having any energy, and this made you more likely to sit at home, “*feeling sorry for yoursel*f” *(participant* *5)*, affecting the mental health and well-being in a negative way.

On the other hand, a participant who was physically well mentioned that this was an important facilitator for her well-being: *“I have no reason to complain, and this has something to do with the fact that I am pretty fit. I can take care of myself, take a walk when I want and so on, and that is nice, and I am not dependent of others” (participant* *3)*.

*(IV) Activities*

I do a lot of things. I don’t know if it is to just forget about it, but I don’t think you should just sit at home and be bored and sad. And I think, that every time you talk to someone, no matter what you talk about, then it helps. But also, just getting outside, take a walk, or a trip on the bicycle, that is also nice (participant 9).

Participating in activities was mentioned as an important facilitator of well-being not only in widowhood but even before the death of the spouse during a long course of illness. Participating in activities was said to help keep up one’s spirits, as “*it doesn’t help if you just sit and feel sorry for yourself all the time. The mood doesn’t get better from that*” *(participant* *5).* Activities mentioned were physical activities such as walking groups and gymnastics, as well as social activities. The structure of the social activities varied depending on where the participants lived. One highlighted, that they had a monthly morning assembly in the local senior citizens centre with singing and coffee. Others mentioned that the senior citizen house in their community had such morning assemblies everyday with breakfast and coffee available. Also volunteering in social activities, such as being a part of the group who made coffee for the morning assemblies, as well as volunteering in various kinds of executive committees and clubs was an important way of keeping one’s mind busy.

It was mentioned that having activities in the community was important, as physical health problems could be a barrier for participating if activities were not nearby. It was furthermore mentioned by several participants, that especially the summertime, weekends and holidays were difficult because most of the activities were on a break, which made time seem longer: *“There are just not so many of these things now that it is summer. Some says that it is much easier now that the weather is fine, and we can go outside and it kind of is. But I would almost say that I feel lonelier now, because there is nothing you can do. Everything just stops now for 3–4 months” (participant 4).*

*(V) Social relations*

I have realized more and more just how important family and good friends are (participant 1).

All participants mentioned the importance of social relations, that is, family, friends, and neighbours. Social relations were important both during the course of illness before death of the spouse, immediately after spousal death, and during widowhood. The importance of social relations was mentioned regarding practical and emotional support.

Several participants mentioned the need for help doing practical things. One widow who did not have kids of her own living nearby expressed, “*my husband had two kids (.) but it is not the same. Yes, they are visiting me, and they are kind and stuff, but they are not so keen on helping*” *(participant* *5).* Having family nearby was mentioned as important for some participants because they could easily come around, if the participant needed help. On the contrary, a participant who did not mention any need for practical support, stated that having frequent phone and video-calls compensated for the distance to his family sufficiently.

Having good friends and neighbours in the community was furthermore important, as they were someone to share activities with and could also help with practical things.

Furthermore, family and friends were mentioned as being important as emotional support. One widow expressed thoughts about wanting to move nearer her family, whereas another stated, that she did not want to move to another town because of her social relations in the community, despite her kids not living nearby. The participants did not express differences in the emotional support received from family and friends, respectively. In contrary, the important factor in terms of emotional support was talking to someone who had known the deceased. Some expressed that they tried to protect their kids from their worries, because they had also lost a parent.

Most participants expressed worries of being a burden to family and friends. They mentioned that having to be the one to initiate contact made them feel uncomfortable, as they worried that they were intrusive. Some stated that they were disappointed of some friends as well as the family of the deceased because they did not reach out. One widow expressed that it made her feel as if she was not only bereaved from her spouse but also his friends and family: *“I have also lost his family. I have no contact to them, so to say. You get tired from always calling and being the one reaching out (…) Yes, so it hurts me, that they are not there for me just slightly, my husband’s family” (participant 6).*

Several participants mentioned their experiences participating in bereavement support groups facilitated by patient support groups or the local churches. The participants expressed different experiences of these groups. One mentioned that listening to the stories of others was overwhelming when you are filled up with your own loss. Another mentioned that she was not comfortable opening up to so many people. A third mentioned, that participating in the bereavement support group was helpful while it lasted and speaking to like-minded was important but: “*you could have imagined that they were someone you would continue seeing or such, but it wasn’t like that at all” (participant 5)*.

The general practitioner and a psychologist were mentioned as valuable emotional support managing widowhood. One pointed out that speaking to a professional was valuable because they did not know the deceased and were not emotionally engaged themselves: “*just talking to someone, who is not emotionally engaged in any way. It is as if it is better to explain it to someone who doesn’t know. I mean, others … even if they were not the closest friends, they still knew him” (participant 6).*

*(VI) Practicalities*

You have the complete responsibility for everything, whereas you had divided duties and responsibility before (…) that is a little hard to get used to (participant 8).

The final category was practicalities. Practicalities were an issue both before and after spousal death. The many practical things to take care of just after the spouse had died such as the funeral were mentioned by almost all participants. Having to clean up and remove the deceased spouse’s stuff such as clothing was a huge task. Some stated that this was chaotic and that they needed huge support from friends and family to take care of this.

Furthermore, dealing with the practical things around the household after the husband’s death was mentioned by some of the widows as a big issue, for an example having to take care of the practical tasks which the deceased spouse used to do and being alone with all the responsibility was a burden. Furthermore, economy was an issue of concern for some widows because it was the deceased husband who took care of this. Economy was also mentioned as a concern for the future. One widow further elaborated this, explaining “*I actually have the same expenses as when we were two*” *(participant* *4).*

On the contrary to these issues of practicalities, it was mentioned that having been able to talk about death with the deceased and having taken care of practicalities such as selling the house was a burden off the widowed persons’ shoulders. Some participants on the other hand mentioned that having something practical to do made things easier in the beginning, and that it was not until this was over, that widowhood became difficult.

Similarly, a widow explained that having shared the responsibility of the economy and the practicalities around the household while the husband lived made it easier for her to manage widowhood: *“there are so many things we do and have done together and talked about and such things and even now in the end, I don’t feel like there was anything that I did not master and were a part of, and I think that is a huge advantage” (participant 3).*

Two out of the three widowers mentioned that having a healthy economy made things easier for them.

### Quantitative analysis

#### Sample characteristics

A total number of 1,095 participants from the Lundby study were included. Of these 16.3% (n = 179) were widowed. There were significantly more women (n = 144, 80.5%) in the widowed group than the married group (n = 457, 49.9%) (p < 0.001). The widowed people were significantly older than the married people with a mean age of 75.8 years (SD 0.75) versus 62.8 years (SD 0.32) in the married group (p < 0.001) and were more often retired (including early retirement) than the married (p < 0.001). Furthermore, they were more often of lower socioeconomic status than the married (blue-collar vs. white collar, p < 0.001). Missing data varied between 7.8% and 28.5% in the different measures. Married participants had most missing data.

#### Quantitative proxies for qualitative experiences of widowhood

We identified questions in the 1997 Lundby questionnaire that served as quantitative proxies for the qualitative categories and codes described above. It was not possible to examine all categories and codes quantitatively as they were not all covered in the Lundby study. The questions identified in the Lundby study and the qualitative category for which they served as proxies are presented in Supplementary File 2.

#### Quantitative associations by marital status

We examined how the quantitative proxies for the qualitative categories and codes, representing the experiences described by the widowed people, were distributed in married and widowed people, respectively (see Table III). We found evidence that some of the experiences described by the widowed people in the qualitative interviews, were more frequent in widowed people compared to married people according to the quantitative proxies in the Lundby Study.

**Table III.** Categories of experiences (listed as I–VI) reported by widowed people in the qualitative interviews. the occurrence of these experiences is quantitatively examined in widowed people compared to married people in a population-based sample by using quantitative proxies for the qualitative categories (I–IV)

	Widowed	Married	*p*-Value*
I. Circumstances around spousal death:			
Spouse has been ill since last follow-up** (n, %)			<0.001^1^
Yes	132 (86.3)	446 (601)	
No	21 (13.7)	296 (39.9)	
II. Mental Health and Well-being			
Crisis or adversity since last follow-up**(n, %)			<0.001^1^
Yes	113 (69.8)	272 (35.2)	
No	49 (30.3)	501 (64.8)	
Mental Health Problems since last follow-up** (n, %)			=0.001^1^
Yes	61 (37.0)	191 (24.7)	
No	104 (63.0)	582 (75.3)	
Suicidal thoughts since last follow-up** (n, %)			=0.117^1^
Yes	12 (8.0)	33 (4.8)	
No	138 (92.0)	653 (95.2)	
Quality of sleep at the moment (n, %)			<0.001^2^
Good	81 (51.3)	497 (68.1)	
Fairly good	40 (25.3)	156 (21.4)	
Poor	37 (23.4)	77 (10.6)	
Satisfied with life at the moment (n, %)			<0.001^2^
Good	108 (67.5)	655 (84.3)	
Fairly good	43 (26.9)	94 (12.1)	
Poor	9 (5.6)	28 (3.6)	
Feels as vital as peers (n, %)			=0.664^2^
Feels more vital than peers	53 (35.8)	216 (33.0)	
Feels just as vital as peers	78 (52.7)	366 (55.9)	
Feels less vital than peers	17 (11.5)	73 (11.2)	
Feels restless (n, %)			=0.800^2^
Often	21 (13.5)	91 (12.4)	
Sometimes	33 (21.2)	184 (25.0)	
Rarely or never	102 (65.4)	462 (62.7)	
Tire easily (n, %)			<0.001^2^
Often	45 (28.5)	126 (17.1)	
Sometimes	42 (26.6)	159 (21.6)	
Rarely or never	71 (44.9)	452 (61.3)	
Feels nervous (n, %)			=0.945^2^
Often	18 (11.3)	82 (11.1)	
Sometimes	18 (11.3)	91 (12.3)	
Rarely or never	123 (77.4)	569 (76.7)	
Feels lonely (n, %)			<0.001^2^
Often	39 (24.7)	26 (3.5)	
Sometimes	43 (27.2)	69 (9.4)	
Rarely or never	76 (48.1)	641 (87.1)	
Cries easily (n, %)			=0.072^2^
Often	51 (32.7)	190 (25.6)	
Sometimes	35 (22.4)	172 (23.2)	
Rarely or never	70 (44.9)	380 (51.2)	
Feels forgetful (n, %)			=0.075^2^
Often	36 (22.8)	111 (15.0)	
Sometimes	53 (33.5)	280 (37.7)	
Rarely or never	69 (43.7)	351 (47.3)	
III. Physical health			
Cardiovascular disease (n, %)			<0.001^1^
Yes	90 (50.3)	270 (29.5)	
No	89 (49.7)	646 (70.5)	
Pulmonary disease (n, %)			=0.021^1^
Yes	29 (16.2)	94 (10.3)	
No	150 (83.8)	822 (89.7)	
Cancer (n, %)			<0.001^1^
Yes	24 (13.4)	50 (5.5)	
No	155 (86.6)	866 (94.5)	
Diseases of CNS (n, %)			<0.001^1^
Yes	65 (36.3)	216 (23.6)	
No	114 (63.7)	700 (76.4)	
Infections (n, %)			=0.055^1^
Yes	18 (10.1)	56 (6.1)	
No	161 (89.9)	860 (93.9)	
Other (n, %)			<0.001^1^
Yes	119 (66.5)	423 (46.2)	
No	60 (33.5)	493 (53.8)	
IV. Activities			
Employment status (n, %)			<0.001^1^
Retired	148 (83.2)	374 (47.2)	
Early retirement (disability pension) or unemployed	11 (6.2)	76 (9.6)	
Labour market active	19 (10.7)	342 (43.2)	
Participates in activities			=0.533^1^
None	10 (6.3)	29 (3.8)	
Few	126 (79.8)	613 (80.0)	
Many	22 (13.9)	124 (16.2)	
V. Social relations			
Number of children (mean, SD)	2.17 (0.13)	2.06 (0.05)	=0.352^3^
VI. Practicalities			
Socioeconomic status (n, %)			<0.001^1^
Blue-collar	115 (64.3)	471 (51.4)	
White-collar	33 (18.4)	334 (36.5)	
Self employed	31 (17.3)	111 (12.1)	
Shared responsibility with spouse (n, %)			=0.291^2^
Poor	10 (6.5)	46 (6.2)	
Fairly good	12 (7.8)	100 (13.6)	
Good	132 (85.7)	592 (80.2)	

^1^Chi-squared test, ^2^chi-squared test for trend, ^3^independent samples t-test

*Missing data varied between 7.8%–28.5% (missing data not shown). All comparative analyses were conducted by listwise deletion of missing data.

**Data is from the 1997 Lundby follow-up. The phrasing “since last follow-up” in the questions refers to the previous follow-up in 1972.

Overall, widowed people had more often had an ill spouse than the married people (p < 0.001) and widowed people had more often experienced a crisis or severe adversity since last follow-up (p < 0.001).

Regarding the qualitative category “mental health and well-being”, we found evidence that widowed people scored poorly compared to the married people: Widowed people had more often had mental health problems since last follow-up compared to married people (p = 0.001). They were less satisfied with life (p < 0.001) and were more often lonely (p < 0.001). They had poorer quality of sleep (p < 0.001) and tired more easily than the married people (p < 0.001). There was no evidence of an increased occurrence of suicidal thoughts or attempts in widowed people compared to married people.

Regarding the qualitative category “physical health problems”, we found evidence that widowed people had more often experienced physical health problems than married people. They had more often cardiovascular diseases (p < 0.001), pulmonary diseases (p < 0.05), disease of CNS (p < 0.001), cancer (p < 0.001) and other diseases (p < 0.001).

We found no evidence of differences between married and widowed people in the proxies used for the categories “social relations” or “activities” (see Table III).

## Discussion

The study identified six qualitative categories representing experiences of widowhood expressed by the participants in the interviews. The categories were concerned with the importance of the circumstances around spousal death, mental health, physical health and how this was associated with mental health and well-being in widowhood, as well as the importance of social relations, participating in activities and practicalities as an issue in widowhood. The study furthermore examined the transferability of the findings and how the categories were quantitatively associated with widowhood by comparing the occurrence of these categories in widowed and married people in another sample. The study found quantitative evidence, supporting the qualitative experiences described by the widowed people, that both physical and mental health problems are frequent and important in widowhood.

An overall interesting finding of the qualitative interviews was the importance of the time before spousal death. Although the opening question of all interviews concerned the time after the spouse had passed away all participants started by telling the story concerning the time before spousal death. In general, the most prominent category in the qualitative interviews was the circumstances around spousal death, which took up a lot of time in all interviews. However, the focus around the time before spousal death was not exclusively concerned around the circumstances around the death of the spouse but concerned most of the categories. Participants who had experienced a long course of illness mentioned this as straining, taking all their energy, which made becoming widowed even harder. Experiences of sudden death, however, were also mentioned as difficult because the widowed were not prepared for death and did not get to say goodbye. It was not possible to quantitatively examine the influence of the circumstances around spousal death in the Lundby study as there was no information on the cause of spousal death. Future cohort studies should examine how the circumstances around spousal death are associated with adverse outcomes of health in widowhood. Unexpected death has been suggested to be worse regarding outcomes of grief and mental health (Sasson & Umberson, 2014; Siflinger, 2016). Carr et al. (2001) however found that also deaths that were forewarned more than 6 months ahead were associated with increased anxiety at 6 and 18 months after spousal loss, reflecting a more complex relationship than previously suggested. This is in line with our qualitative findings that both expected and unexpected death affected the outcome of mental health and well-being in widowhood, although the thoughts and symptoms related to the circumstances of the spousal death might be different. Participants who had been able to talk about death with their spouse, as well as participants who had not been able to do this, expressed this as important for their mental health and well-being after the spouse died. Carr et al. similarly found that having discussed death with the spouse was associated with lower levels of intrusive thoughts (Carr et al., 2001).

Widowed people expressed that physical health problems affected the mental health and well-being in widowhood. Our quantitative analysis showed that physical health problems were significantly more common in widowed people than married people for almost all disease categories, except infections. Wilcox et al. (2003) also showed poorer physical health in widowed people compared to married people. Interestingly, they found that this was evident already at baseline (Wilcox et al., 2003). This could indicate that the poorer physical health seen in widowed participants is already present at the time the participant becomes widowed and perhaps even before.

Some participants who have had an ill spouse expressed that they had deliberately postponed taking care of their own health problems, because this was inconvenient during the spouse’s illness. This finding is supported in a previous study of 21 caregivers (DiGiacomo et al., 2013). This could be a part of the explanation for the health disparities by marital status shown previously (Wilcox et al., 2003), which we have also shown in the quantitative examination.

Similarly, widowed people have higher levels of depressive symptoms already before widowhood compared to those who remain married (Sasson & Umberson, 2014). We found in the Lundby sample, that widowed people had significantly more often experienced mental health problems since the last follow-up than the married. This is in line with previous research, showing that widowed people have a high prevalence of depression and anxiety disorders (Kristiansen et al., 2019a, 2019b; Onrust & Cuijpers, 2006), and that depression and depressive symptoms are more common in widowed people than in married people (Schaan, 2013).

In relation to mental health and well-being, the widowed participants in the interviews furthermore expressed difficulties sleeping, restlessness, having problems concentrating and remembering as well as feeling tired. Our quantitative analysis of the Lundby sample showed that these symptoms were more common in the widowed than in the married (although nonsignificant regarding restlessness and forgetfulness). The participants in the qualitative interviews expressed a connection between physical health and mental health and well-being, for an example because physical health problems hindered them participating in social activities, leading to increased loneliness and more time for speculations. Utz et al. similarly found that self-reported physical health was correlated to grief and depressive symptoms (Utz et al., 2012), supporting the experiences by the widowed participants in our interviews. Similar to the study by Wilcox et al. (2003) they did not find significant changes of self-reported health over time but found that baseline health and health during the first months of widowhood were most important for the mental health outcomes (Utz et al., 2012).

### Strengths and limitations

The study used an exploratory sequential mixed methods approach combining a Danish qualitative interview study with a Swedish cohort study. Although we consider the combination of cross-countries qualitative and quantitative data a strength of the study the method also had some limitations. The study used a sequential approach to ensure independency within the two studies. That is, assessment of the quantitative data in the present study was not conducted until the qualitative analysis was completely finished. However, the Lundby study was conducted prior to the qualitative study. Consequently, it was not possible to influence on the questions asked in the Lundby study. Although this is a strength regarding independency of the data it also had the consequences that it was not possible to examine all the findings from the qualitative interviews quantitatively in the Lundby study. Each of the Lundby questions that were used in the quantitative analysis was concerned with the same topic as the category and code for which it served as a proxy. Nonetheless, we could not influence on the phrasing of the questions, and thus, although they served as useful proxies, the questions were not exactly the same as what has been mentioned by the widowed participants in the qualitative interviews. Although this does limit the possibilities for us to verify all our qualitative findings quantitatively, this also ensures independency within the two studies, strengthening the implications of any association found and thereby the trustworthiness of the study, as the findings are consistent despite the two studies being conducted in two different countries (although similar in many ways) and almost 20 years apart.

A limitation of the study is missing data. For the measures examined the distribution of missing data varied from 7.8% to 28.5%. Missing data were not evenly distributed by marital status and was for most measures highest in the married group. Although this indicates that data are not missing at random, comparative analyses have been performed by listwise deletion of missing data. We have no reason to believe that marital status itself influenced on whether or not participants answered the questions in the Lundby study, as data were not gathered in relation to marital status. An explanation could be that people who experienced negative outcomes regarding a question were more likely to answer the question than people who did not experience the phenomena in question. As widowed participants did score more negatively in most measures examined, this could explain some of the disparity in the distribution of missing data.

Also, the qualitative part of the study has some strengths and limitations. All interviews were conducted by the first author. Her experience and interest within mental illness as well as preunderstanding of adverse effects of widowhood might have impacted the interviews. This was sought minimized by keeping the opening question open ended and generally following the story the participants decided to tell, ensuring a rich description from the participants’ own perspectives (Kvale & Brinkmann, 2009).

Furthermore, the analysis was partly iterative, that is, interviews were read and discussed between two investigators with different experiences and preunderstandings and themes identified in this process were sought explored in the subsequent interviews. Although the iterative analysis could potentially have steered the interviews in a certain direction, the interviewer ensured to only ask elaborately if the themes were mentioned by the participants themselves or could be introduced as a natural part of the conversation for an example if the participant did not have more to tell themselves. As such, asking about these themes did not hinder the participants from sharing their own perspectives first and did therefore not influence the reaching of data saturation. On the other hand, the iterative analysis strengthened the credibility of the analysis, as discussing the interviews increased the self-awareness of the interviewer in terms of the influence of her preunderstanding and possible biases due to this (Elo et al., 2014).

Finally, the investigators participating in the analysis had different backgrounds, experiences and preunderstandings, and the coding and abstraction was conducted while discussed by all three investigators ensuring different perspectives, while keeping a descriptive focus as close to the participants’ own words and phrasings as possible (Chafe, 2017), in order to ensure conformability of the findings (Elo et al., 2014).

The participants in the qualitative part of the study were purposeful sampled, in order to have different experiences and perspectives on becoming widowed. Therefore, there were no specific inclusion criteria except age, duration of widowhood, and being able to speak Danish. We consider this a strength of the study. Nonetheless, the sampling method also has some limitations. First, defining that the persons had to be widowed for less than two years is likely to have influenced the findings. Studies have shown that although it remains high for up to ten years of widowhood, adverse effects on mental health decreases over time (Kristiansen et al., 2019b) Therefore, the experiences described by the participants regarding for an example mental health and well-being might have been different if the study included people who had been widowed for longer periods. Similarly, some of the participants were recruited through care contacts with their general practitioner, and some through contact with the psychiatric facility. Others were recruited from the community. Even though none of the contacts was due to a bereavement-related issue, it could still be that the participants that had been in contact with their general practitioner or the psychiatric facility were more severely affected by their grief than the participants who were recruited from the community—this was however not evident to the interviewer, who on the contrary experienced a variety of the overall experiences described not depending on the recruitment method. As such, we consider using different settings for recruitment a strength of the study, as this ensures a more varied picture of the experiences of widowhood.

Similarly, a major strength of the Lundby study is that the study is population based, examining traits and adverse outcomes in a non-patient population, making it representative of the background population (Henderson & Jablensky, 2010; Nettelbladt et al., 2005). Thus, the results of this study are not patient specific or restricted to certain circumstances of becoming widowed as they reflect general experiences of widowhood in a community setting. Furthermore, the verification, that at least some of the experiences expressed in the qualitative interviews, also occur in widowed people in a different sample, in a different point in time and even another country makes it reasonable to make more general conclusions based on the findings. As such, we believe the data show both high dependability as well as transferability, contributing to high trustworthiness of the study (Elo et al., 2014). We consider this is a major strength of our study.

### Implications of the findings

The study showed that there is an association between physical and mental health in widowhood. As previous studies suggest that widowed people already have poorer health outcomes prior to becoming widowed (Wilcox et al., 2003) this association calls for further attention. Our finding that the widowed participants experience the circumstances around the spousal death as well as the time before spousal death in general to be important for their later well-being could be a possible pathway into understanding some of the adverse effects of health seen in widowhood. This is an important finding, and future studies should focus on examining the influence of the time before spousal death and the circumstances around spousal death on mental and physical health outcomes in widowhood.

## Conclusion

The experiences of widowhood were concerned around six categories including circumstances around spousal death, mental health and well-being, physical health, activities, practicalities and social relations, of which the circumstances around spousal death seemed to be the most prominent. In general, the time before spousal death was important to how one experienced being widowed. This is important as future studies and interventions should focus not only on the time after spousal death but begin already before the loss of the spouse.

Being physically ill negatively affected mental health and well-being in widowhood, partly because it affected the ability to participate in activities and social relations. This is important, as both physical and mental health problems are frequently occurring and are more common in widowed people than married people.

## Supplementary Material

Supplemental Material

## References

[cit0001] Anusic, I., & Lucas, R. E. (2014). Do social relationships buffer the effects of widowhood? A prospective study of adaptation to the loss of a spouse. *Journal of Personality*, 82(5), 367–16. 10.1111/jopy.12067 24033325 PMC3936003

[cit0002] Blanner Kristiansen, C., Juel, A., Vinther, H. M., & AM, H. (2015). Promoting physical health in severe mental illness: Patient and staff perspective. *Acta Psychiatrica Scandinavica*, 132(6), 470–478. 10.1111/acps.12520 26696384

[cit0003] Blanner, C., Mejldal, A.., Prina, A.M., Munk-Jørgensen, P., Ersbøll, A. K., & Andersen, K. (2020). Widowhood and mortality: A Danish nationwide register-based cohort study. Epidemiology and Psychiatric Sciences, 29(29), e149. 10.1017/S2045796020000591 PMC7443807 32744212

[cit0004] Bradshaw, C., Atkinson, S., & Doody, O. (2017). Employing a qualitative description approach in health care research. *Global Qual Nurs Res*, *24(**4**)*, DOI: 10.1177/2333393617742282. PMC5703087 29204457

[cit0005] Butler, A. E., Copnell, B., & Hall, H. (2019). Researching people who are bereaved: Managing risks to participants and researchers. *Nursing Ethics*, 26(1), 224–234. 10.1177/0969733017695656 28367685

[cit0006] Carr, D. (2020). Mental health of older widows and widowers: Which coping strategies are most protective? *Aging Ment Health, 24*(2), 291-299. DOI: 10.1080/13607863.2018.1531381. Epub 2018 Dec 27. 30588820

[cit0007] Carr, D., House, J. S., Wortman, C., Nesse, R., & Kessler, R. C. (2001). Psychological adjustment to sudden and anticipated spousal loss among older widowed persons. *The Journals of Gerontology Series B: Psychological Sciences and Social Sciences*, 56(4), S237–S248. 10.1093/geronb/56.4.S237 11445616

[cit0008] Chafe, R. (2017). The value of qualitative description in health services and policy research. *Healthc Policy*, 12(3), 12–18. https://www.ncbi.nlm.nih.gov/pmc/articles/PMC5344360/. 28277201 PMC5344360

[cit0009] Daatland, S. O. (1994). Stress and strategies in care systems: The Scandinavian experience. *Social Science & Medicine*, 38(7), 867–874. 10.1016/0277-9536(94)90420-0 8202736

[cit0010] de Vries, B., Rebecca., U., Caserta, M., & Lund, D. (2014). Friend and family contact and support in early widowhood. *The Journals of Gerontology Series B: Psychological Sciences and Social Sciences*, 69(1), 75–84. 10.1093/geronb/gbt078 PMC3894122 24170717

[cit0011] DiGiacomo, M., Lewis, J., Nolan, M. T., Phillips, J., & Davidson, P. M. (2013). Transitioning from caregiving to widowhood. *Journal of Pain and Symptom Management*, 46(6), 817–825. 10.1016/j.jpainsymman.2013.01.005 23571208

[cit0012] DiGiacomo, M., Lewis, J., Phillips, J., Nolan, M., & Davidson, P. M. (2015). The business of death: A qualitative study of financial concerns of widowed older women. *BMC Women’s Health*, 18(15), 36. 10.1186/s12905-015-0194-1 PMC4407784 25906773

[cit0013] Einiö, E., & Martikainen, P. (2019). Risk of hospitalization for cancer, musculoskeletal disorders, injuries or poisonings surrounding widowhood. *American Journal of Epidemiology*, 188(1), 110–118. 10.1093/aje/kwy184 30137200

[cit0014] Elo, S., Kääriäinen, M., Kanste, O., Tarja, P., Utrainen, K., & Kyngäs, H. (2014, Jan-March). Qualitative content analysis: A focus on trustworthiness. *SAGE Open*, *4*(1), 1–10. 10.1177/2158244014522633.

[cit0015] Elo, S., & Kyngäs, H. (2008). The qualitative content analysis process. *Journal of Advanced Nursing*, 62(1), 107–115. 10.1111/j.1365-2648.2007.04569.x 18352969

[cit0016] Hardy-Bougere, M. (2008). Cultural manifestations of grief and bereavement: A clinical perspective. *Journal of Cultural Diversity*, 15(2), 66–69. PMID: 18649443. 18649443

[cit0017] Henderson, S., & Jablensky, A. (2010). The Lundby study. *Australian & New Zealand Journal of Psychiatry*, 44(1), 1–3. 10.3109/00048670903393704 20073562

[cit0018] Iqbal, R., & Todi, P. (2015). The Nordic model: Existence, emergence and sustainability. *Procedia Economics and Finance*, 30, 336–351. 10.1016/S2212-5671(15)01301-5

[cit0019] Jaul, E., & Barron, J. (2017). Age-related diseases and clinical and public health implications for the 85 years old and over population. *Frontiers in Public Health*, *1**1*(5):, 335. 10.3389/fpubh.2017.00335 29312916 PMC5732407

[cit0020] Jeon, G.-S., Jang, S.-N., Kim, D.-S., & Cho, S.-I. (2013). Widowhood and depressive symptoms among Korean elders: The role of social ties. *The Journals of Gerontology Series B: Psychological Sciences and Social Sciences*, 68(6), 963–973. 10.1093/geronb/gbt084 24150177

[cit0021] Juel A., Hjorth, P., Munk-Jorgensen, P., & Buus, N (2018). Health Beliefs and Experiences of a Health Promotion Intervention Among Psychiatric Patients With Substance Use: An Interview Study. *Arch Psychiatric Nurs*, 32(3), 462–468. 10.1016/j.apnu.2018.01.004 29784231

[cit0022] Kettles, A. M., Creswell, J. W., & Zhang, W. (2011). Mixed methods research in mental health nursing. *Journal of Psychiatric and Mental Health Nursing*, 18(6), 535–542. 10.1111/j.1365-2850.2011.01701.x 21749560

[cit0023] Kristiansen, C. B., Kjær, J. N., Hjorth, P., Andersen, K., & Prina, A. M. (2019a). Prevalence of common mental disorders in widowhood: A systematic review and meta-analysis. *Journal of Affective Disorders*, *15* 245), 1016–1023. 10.1016/j.jad.2018.11.088 30699843

[cit0024] Kristiansen, C. B., Kjaer, J. N., Hjorth, P., Andersen, K., & Prina, A. M. (2019b). The association of time since spousal loss and depression in widowhood: A systematic review and meta-analysis. *Social Psychiatry and Psychiatric Epidemiology*, 54(7), 781–792. 10.1007/s00127-019-01680-3 30887075

[cit0025] Kvale, S., & Brinkmann, S. (2009). *Interviews: Learning the craft of qualitative research interviewing* (2nd ed.). Sage Publications.

[cit0026] Kyung Do, Y., & Malhotra, C. (2012). The effects of coresidence with an adult child on depressive symptoms among older widowed women in South Korea: An instrumental variables estimation. *The Journals of Gerontology. Series B, Psychological Sciences and Social Sciences*, 67(3), 384–391. 10.1093/geronb/gbs033 22421809

[cit0027] Maciejewski, P. K., Zhang, B., Block, S. D., & Prigerson, H. G. (2007). An empirical examination of the stage theory of grief. *JAMA*, 297(7), 716–723. 10.1001/jama.297.7.716 17312291

[cit0028] Moon, J. R., Kondo, N., Glymour, M. M., & Subramanian, S. V. (2011). Widowhood and mortality: A meta-analysis. *PloS One*, 6(8), e23465. 10.1371/journal.pone.0023465 21858130 PMC3157386

[cit0029] National Committe on Health Research Ethics. (2019). What to notify? Retrieved November 1, 2019, from http://en.nvk.dk/how-to-notify/what-to-notify

[cit0030] Neergaard, M. A., Olesen, F., Andersen, R. S., & Sondergaard, J. (2009). Qualitative description - The poor cousin of health research? *BMC Medical Research Methodology*, 9(1), 52. 10.1186/1471-2288-9-52 19607668 PMC2717117

[cit0031] Nettelbladt, P., Bogren, M., Mattisson, C., Ojesjo, L., Hagnell, O., Hofvendahl, E., Toråker, P., & Bhugra, D. (2005). Does it make sense to do repeated surveys? – the Lundby Study, 1947-1997. *Acta Psychiatrica Scandinavica*, 111(6), 444–452. 10.1111/j.1600-0447.2005.00518.x 15877711

[cit0032] Nseir, S., & Larkey, L. K. (2013). Interventions for spousal bereavement in the older adult: An evidence review. *Death Studies*, 37(6), 495–512. 10.1080/07481187.2011.649941 24520924

[cit0033] O’Rourke, N. (2004). Cognitive adaptation and women’s adjustment to conjugal bereavement. *Journal of Women & Aging*, 16(1–2), 87–104. 10.1300/J074v16n01_07 15149926

[cit0034] Onrust, S. A., & Cuijpers, P. (2006). Mood and anxiety disorders in widowhood: A systematic review. *Aging & Mental Health*, 10(4), 327–334. 10.1080/13607860600638529 16798624

[cit0035] Pedersen, K. M., Andersen, J. S., & Sondergaard, J. (2012). General practice and primary health care in Denmark. *The Journal of the American Board of Family Medicine*, 25(Suppl 1), S34–38. 10.3122/jabfm.2012.02.110216 22403249

[cit0036] Sandelowski, M. (2000). Whatever happened to qualitative description? *Research in Nursing & Health*, 23(4), 334–340. 10.1002/1098-240X(200008)23:4<334::AID-NUR9>3.0.CO;2-G 10940958

[cit0037] Sasson, I., & Umberson, D. J. (2014). Widowhood and depression: New light on gender differences, selection, and psychological adjustment. *The Journals of Gerontology Series B: Psychological Sciences and Social Sciences*, 69(1), 135–145. 10.1093/geronb/gbt058 PMC3894126 23811294

[cit0038] Schaan, B. (2013). Widowhood and depression among older Europeans – The role of gender, caregiving, marital quality, and regional context. *The Journals of Gerontology Series B: Psychological Sciences and Social Sciences*, 68(3), 431–442. 10.1093/geronb/gbt015 PMC3627659 23591571

[cit0039] Schut, H., & Stroebe, M. S. (2005). Interventions to enhance adaptation to bereavement. *Journal of Palliative Medicine*, 8(Suppl 1), S140–147. 10.1089/jpm.2005.8.s-140 16499462

[cit0040] Shear, K. (2015). Complicated grief. *New England Journal of Medicine*, 372(2), 153–160. 10.1056/NEJMcp1315618 25564898

[cit0041] Shor, E., Roelfs, D. J., Curreli, M., Clemow, L., Burg, M. M., & Schwartz, J. E. (2012). Widowhood and mortality: A meta-analysis and meta-regression. *Demography*, 49(2), 575–606. 10.1007/s13524-012-0096-x 22427278 PMC3640496

[cit0042] Siflinger, B. (2016). The effect of widowhood on mental health – An analysis of anticipation patterns surrounding the death of a spouse. *Health economics*, 26(12), 1505–1523. 10.1002/hec.3443 27747997

[cit0043] StataCorp. (2019). *Stata Statistical Software: Release 16*.

[cit0044] Statistics Denmark – Statsbank. Retrieved November 1, 2019, from https://www.statistikbanken.dk/

[cit0045] Stroebe, M., Stroebe, W., Schut, H., & Boerner, K. (2017). Grief is not a disease but bereavement merits medical awareness. *Lancet*, 389(10067), 347–349. 10.1016/S0140-6736(17)30189-7 28137681

[cit0046] Stroebe, M., Stroebe, W., Schut, H., & Boerner, K. (2017). Grief is not a disease but bereavement merits medical awareness. The Lancet, 389(10067), 347–349 doi:10.1016/S0140-6736(17)30189-7 28137681

[cit0047] The Loomba Foundation. (2015). The Global Widows Report 2015. A global overview of deprivation faced by widows and their children. Retrieved November 1, 2019, from https://www.theloombafoundation.org/sites/default/files/2019-06/WWR.pdf

[cit0048] Utz, R. L., Caserta, M., & Lund, D. (2012). Grief, depressive symptoms, and physical health among recently bereaved spouses. *Gerontologist*, 52(4), 460–471. 10.1093/geront/gnr110 22156713 PMC3391379

[cit0049] Valdimarsdottir, U., Helgason, A. R., Fürst, C.-J., Adolfsson, J., & Steineck, G. (2003). Long-term effects of widowhood after terminal cancer: A Swedish nationwide follow-up. *Scandinavian Journal of Public Health*, 31(1), 31–36. 10.1080/14034940210165109 12623522

[cit0050] van den Berg, G. J., Lindeboom, M., & Portrait, F. (2011). Conjugal bereavement effects on health and mortality at advanced ages. *Journal of Health Economics*, 30(4), 774–794. 10.1016/j.jhealeco.2011.05.011 21715034

[cit0051] Weiser, P., Becker, T., Losert, C., Alptekin, K., Berti, L., Burti, L., Burton, A., Dernovsek, M., Dragomirecka, E., Freidl, M., Friedrich, F., Genova, A., Germanavicius, A., Halis, U., Henderson, J., Hjorth, P., Lai, T., Larsen, J. I., Lech, K., Lucas, R., & Kilian, R. (2009). European network for promoting the physical health of residents in psychiatric and social care facilities (HELPS): Background, aims and methods. *BMC Public Health*, *28* (9), 315. 10.1186/1471-2458-9-315 19715560 PMC2741451

[cit0052] Wilcox, S., Evenson, K. R., Aragaki, A., Wassertheil-Smoller, S., Mouton, C. P., & Loevinger, B. L. (2003). The effects of widowhood on physical and mental health, health behaviors, and health outcomes: The Women’s Health Initiative. *Health Psychology : Official Journal of the Division of Health Psychology, American Psychological Association*, 22(5), 513–522. 10.1037/0278-6133.22.5.513 14570535

[cit0053] Yaolin., P., Cong, Z., & Wu, B. (2019). Risk and resiliency in the relationship between widowhood and depressive symptoms among older Mexican Americans. *Journal of Cross-Cultural Gerontology*, 34(2), 149–170. 10.1007/s10823-019-09367-7 30903551

[cit0054] Zhou, J., & Hearst, N. (2016). Health-related quality of life of among elders in rural China: The effect of widowhood. *Quality Life Res*, 25(12), 3087–3095. 10.1007/s11136-016-1338-y PMC5102975 27294437

[cit0055] Zisook, S., & Shear, K. (2009). Grief and bereavement: What psychiatrists need to know. *World Psychiatry* 8(2):67–74. 10.1002/j.2051-5545.2009.tb00217.x PMC2691160 19516922

