# Supplementary material for: Experiences of becoming widowed in old age – a cross-countries study with qualitative interviews from Denmark and quantitative measures of association in a Swedish sample
Source: Int J Qual Stud Health Well-being. 2021 Feb 4;16(1):1871181. doi: 10.1080/17482631.2020.1871181 (PMC8725736; doi:10.1080/17482631.2020.1871181)
Supplement: Supplemental Material [file ZQHW_A_1871181_SM0259.docx]

**Supplementary File 1**

**Lundby Questionnaire 1997**


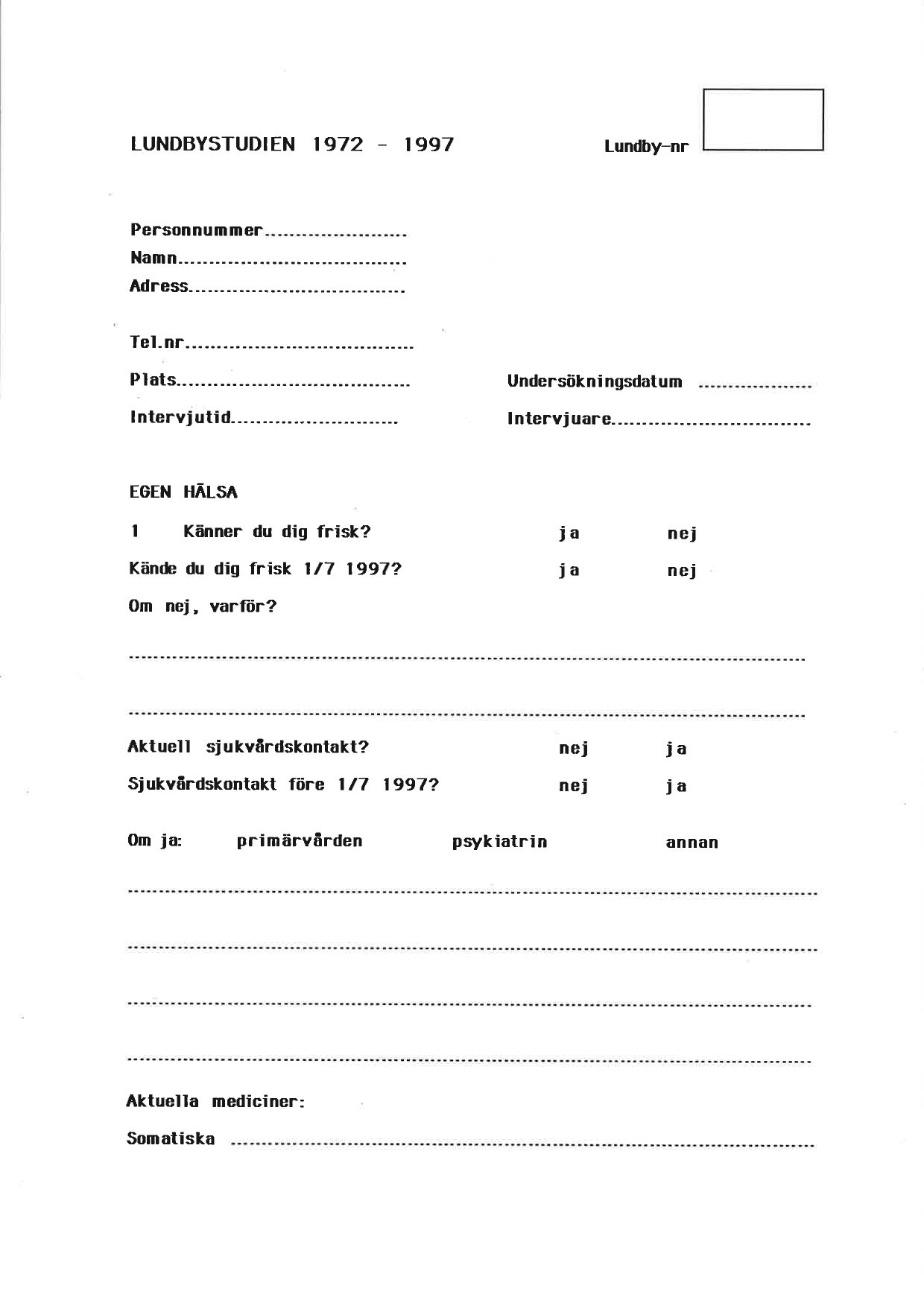


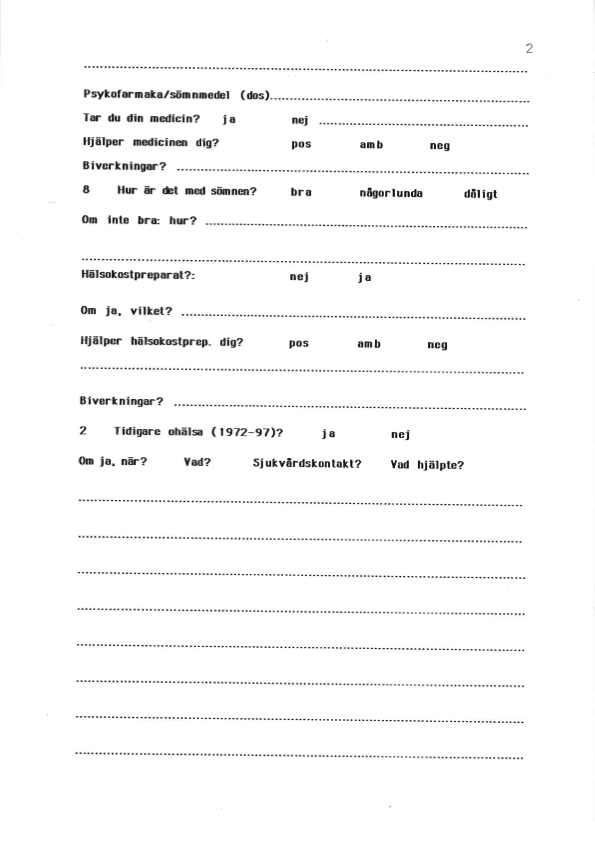

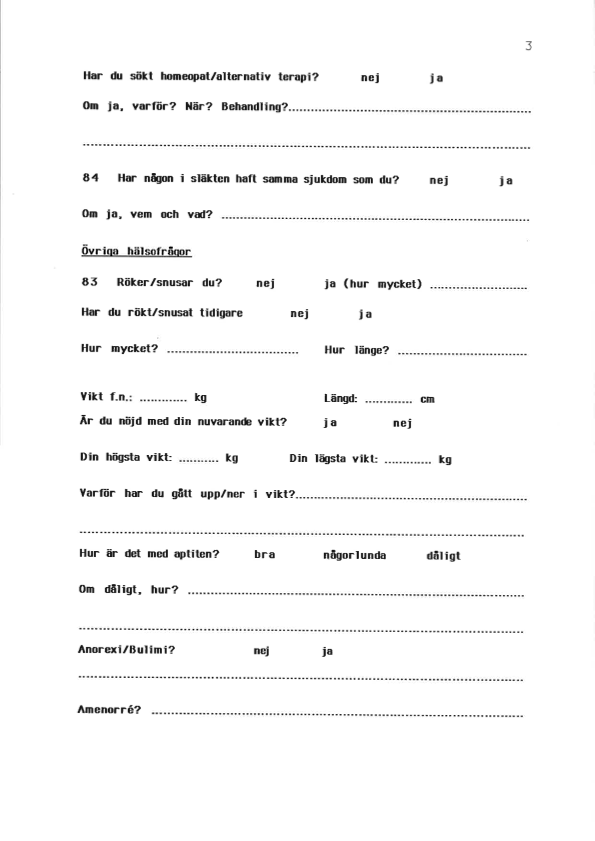

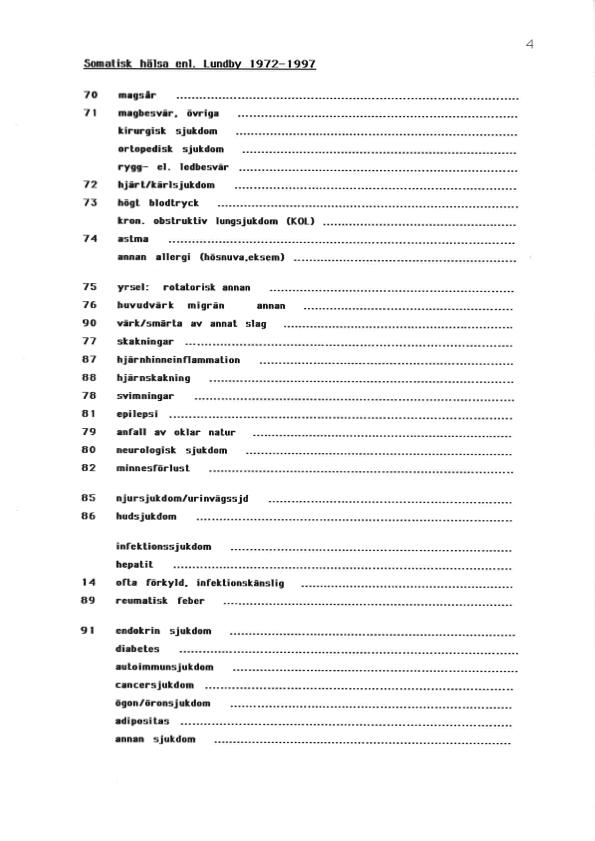

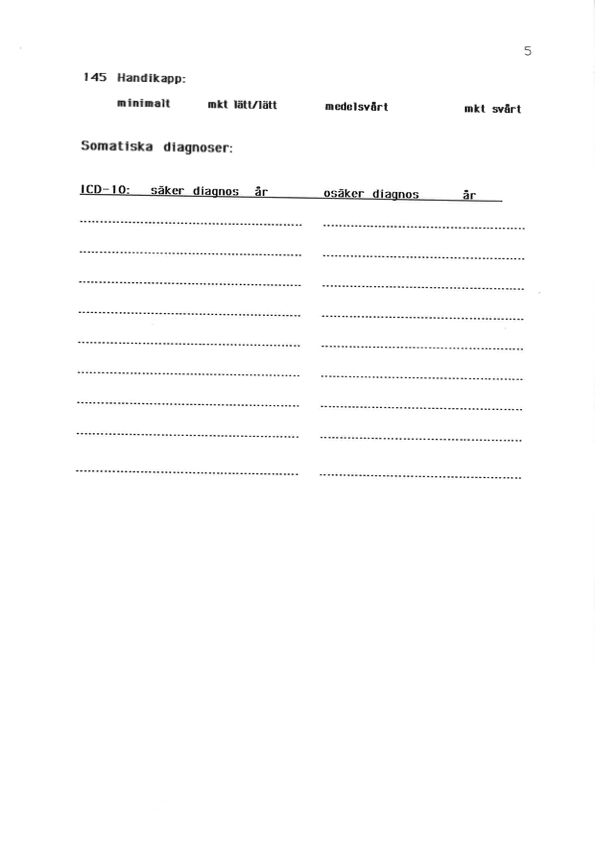

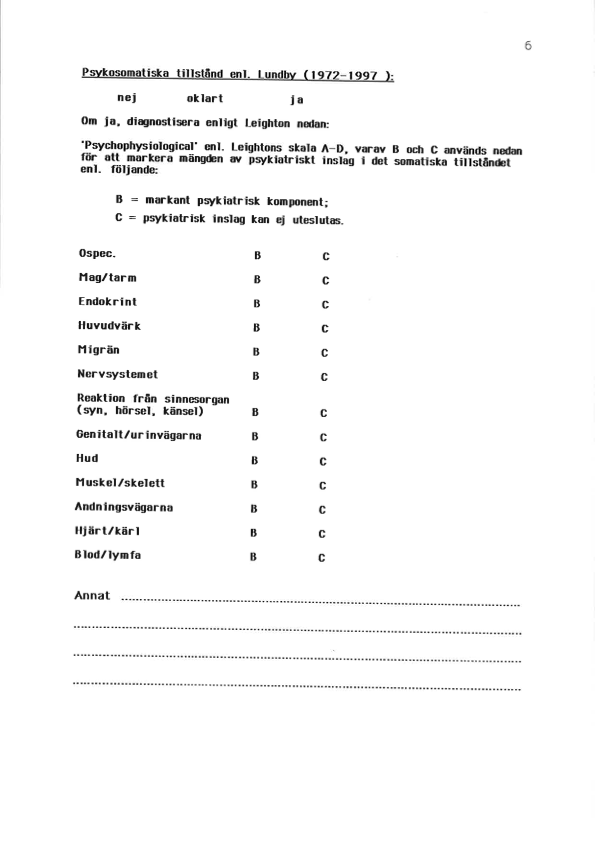

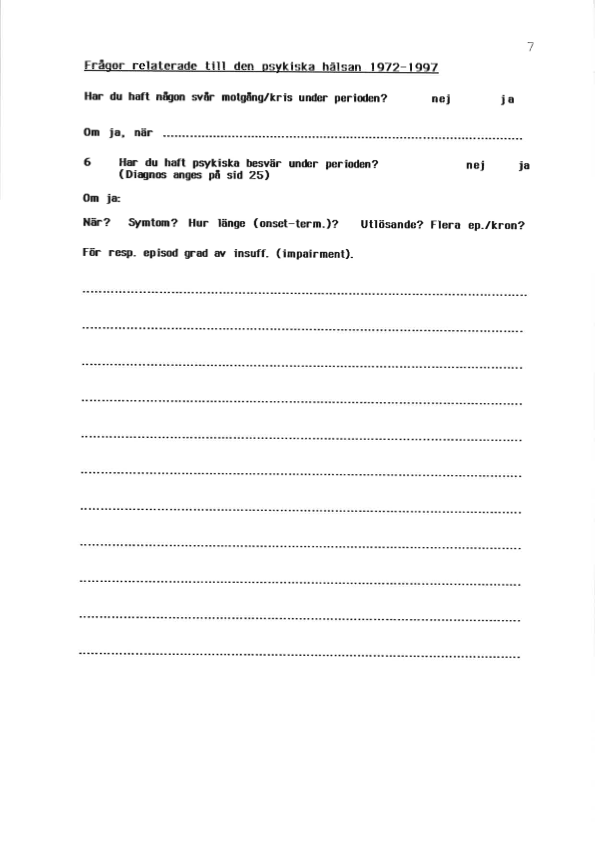

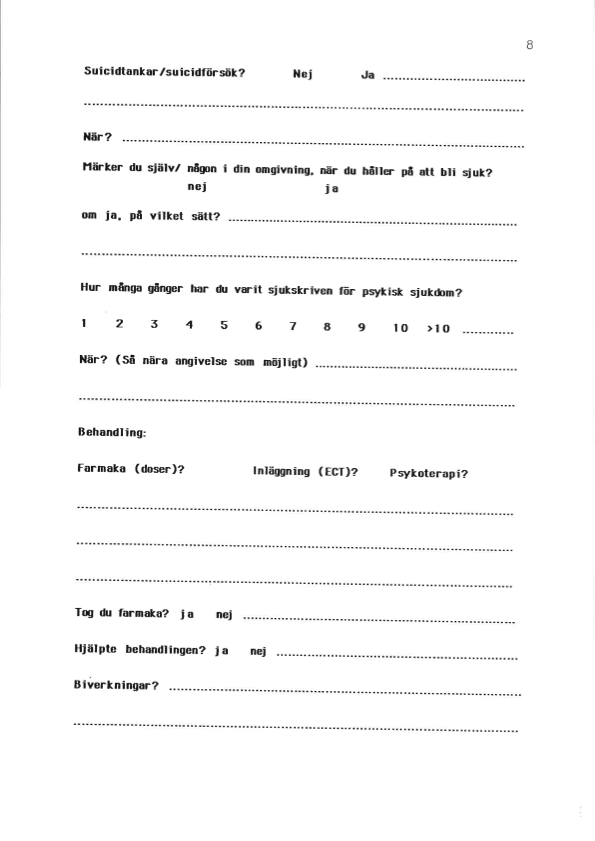

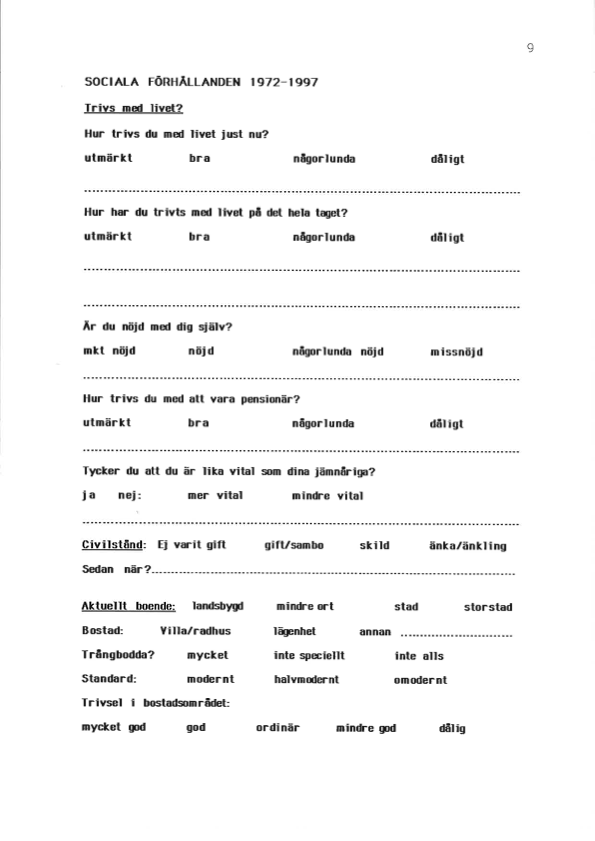

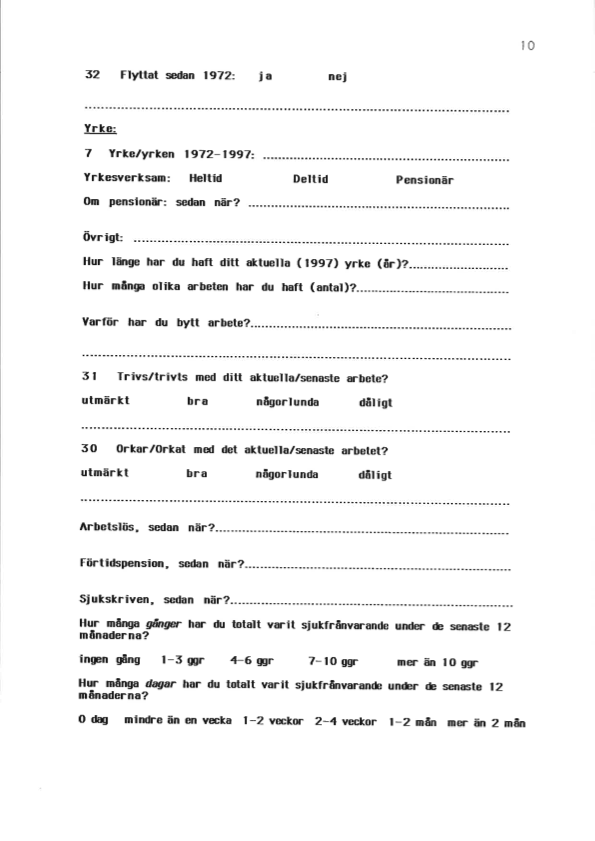

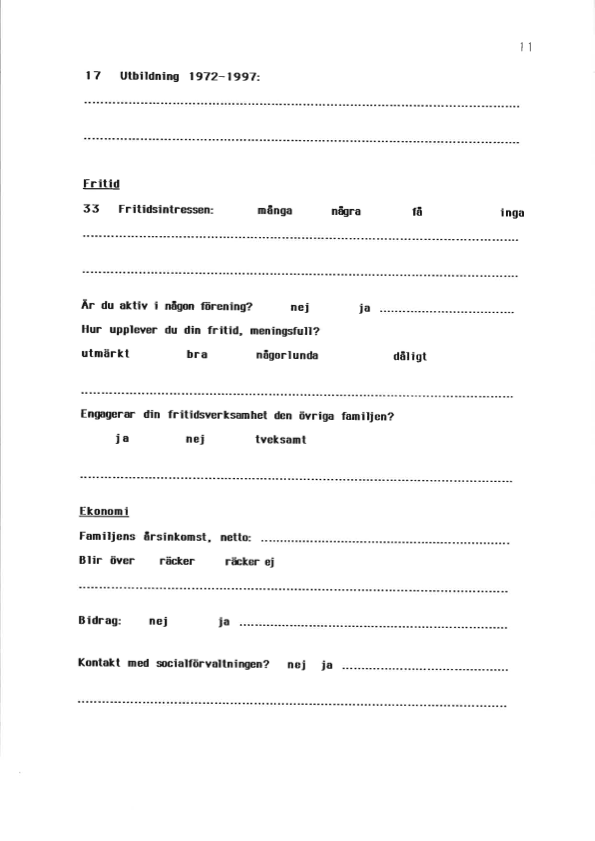

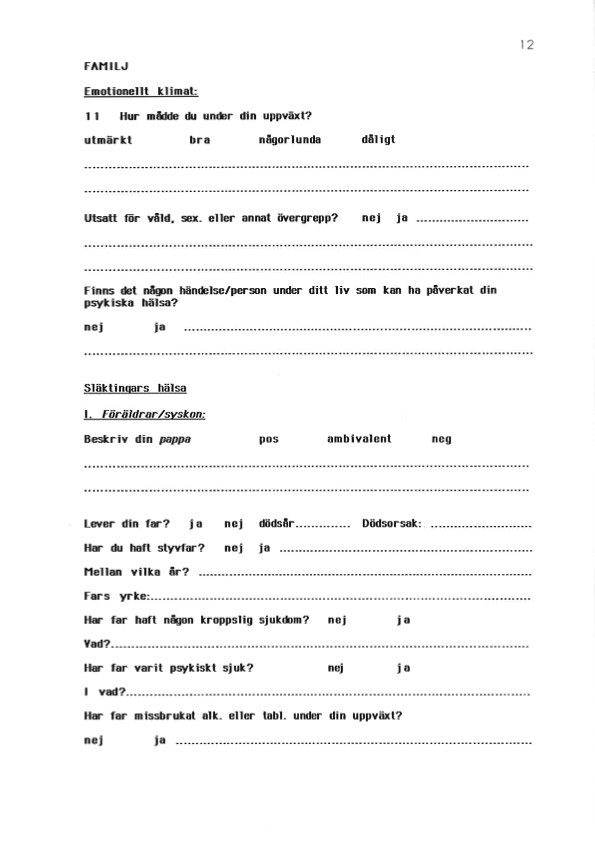

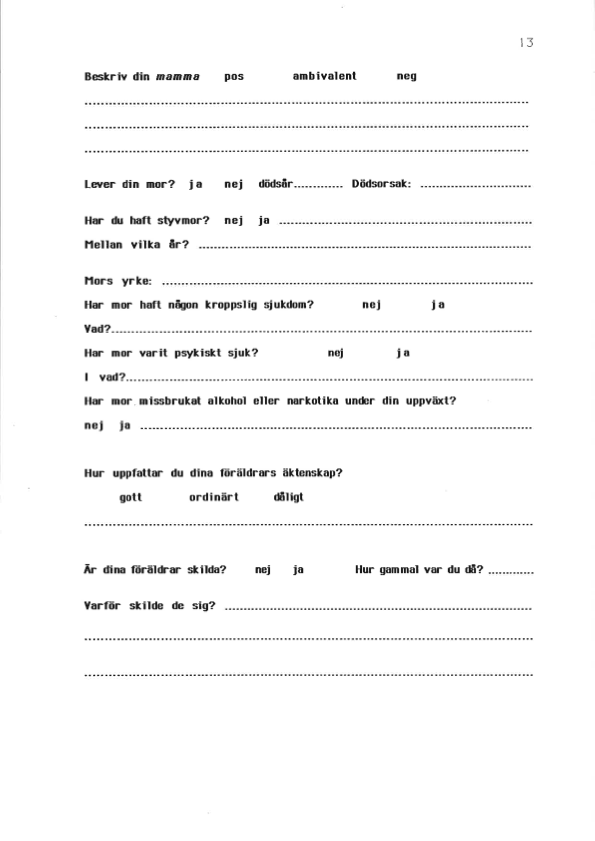

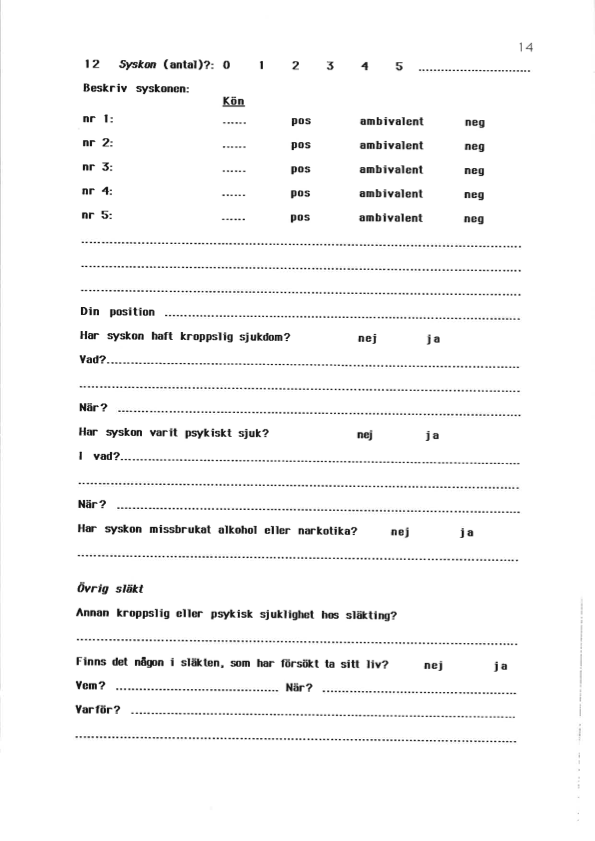

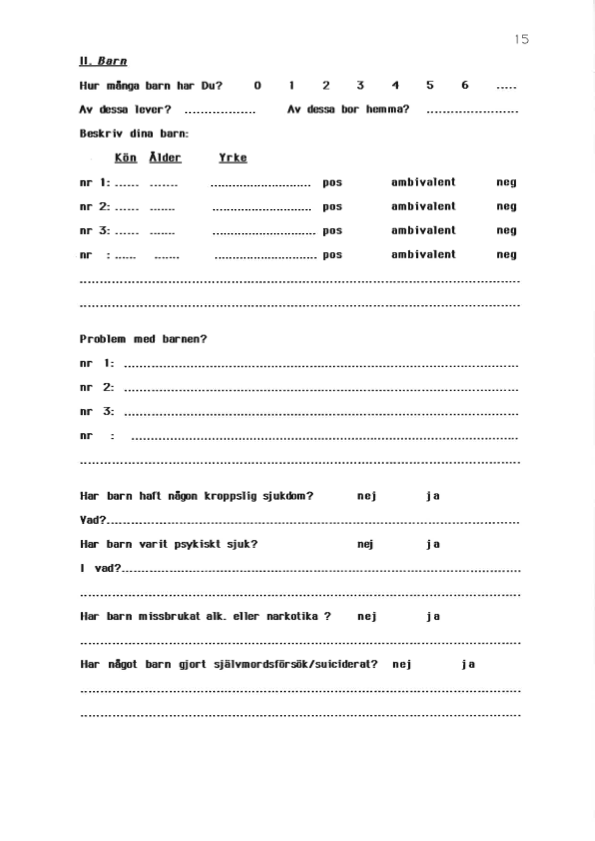

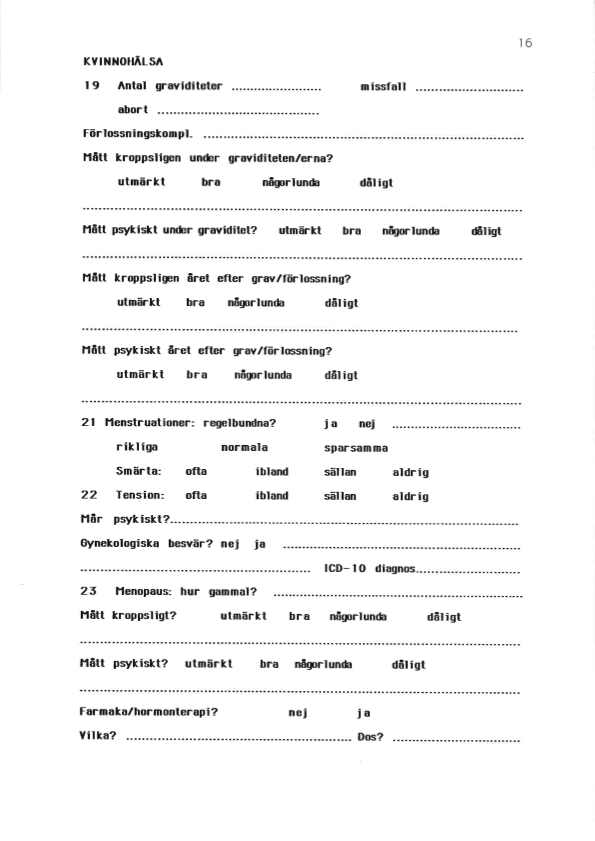

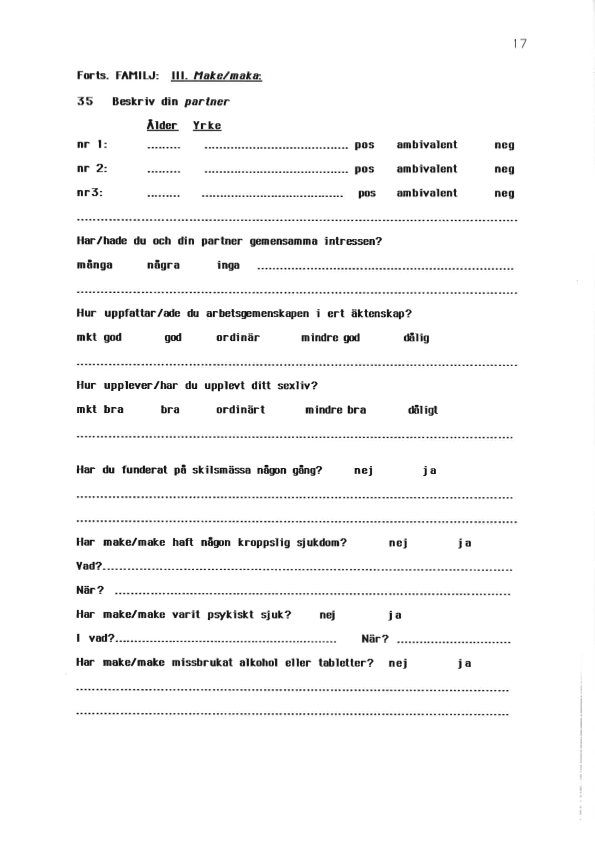

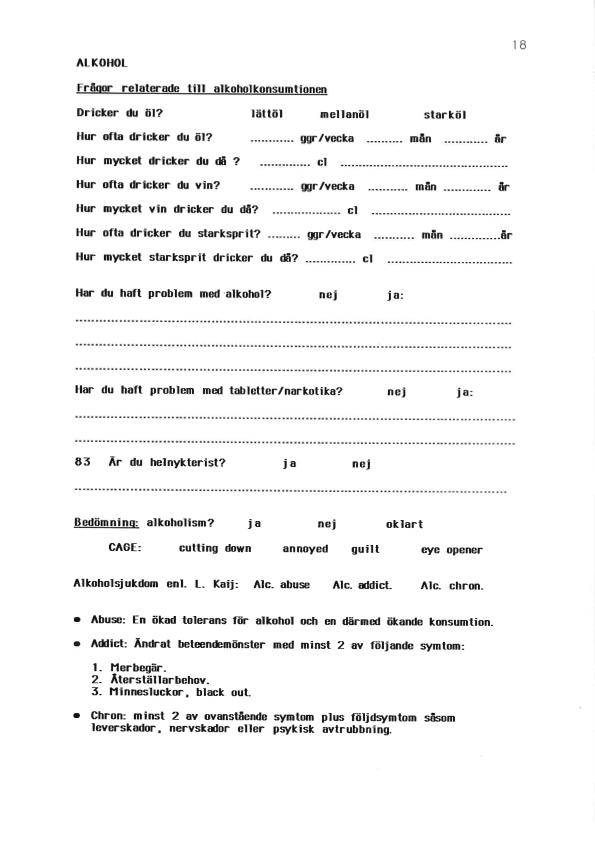

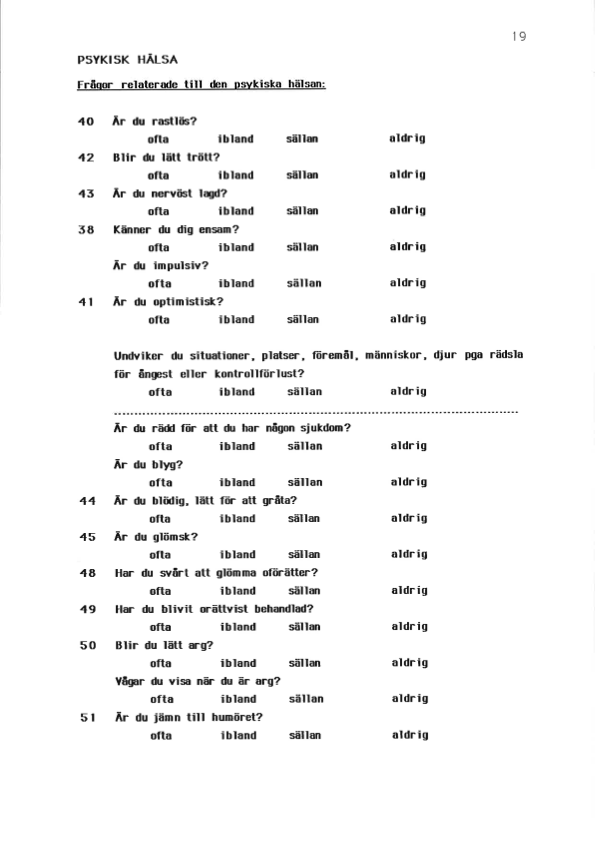

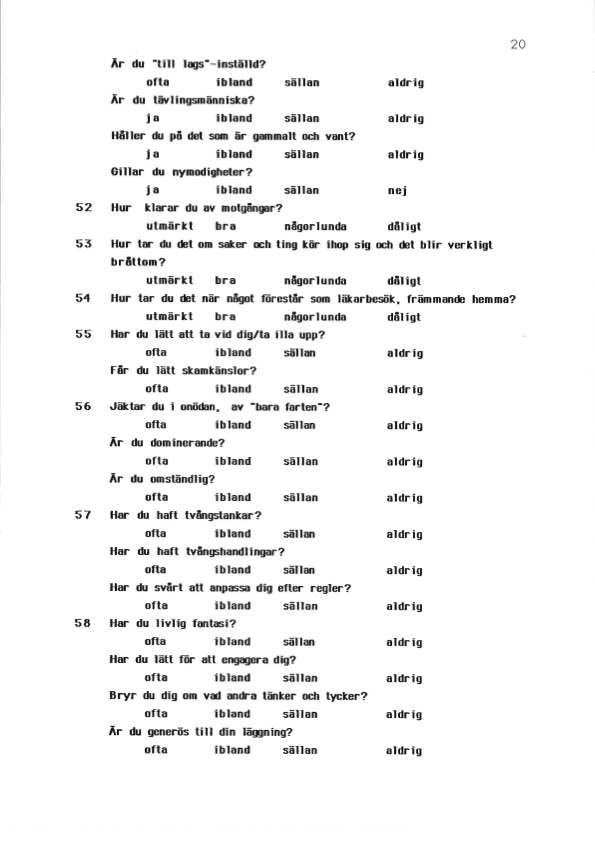

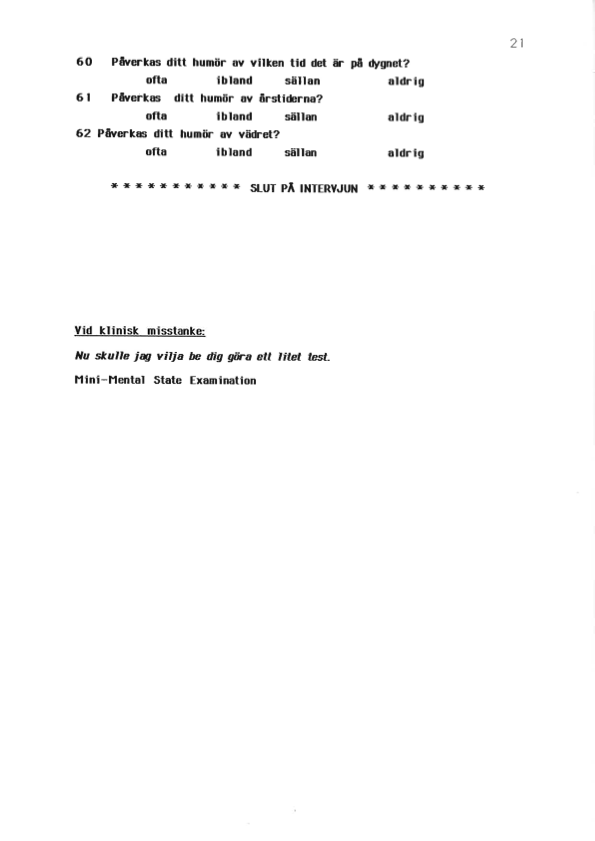

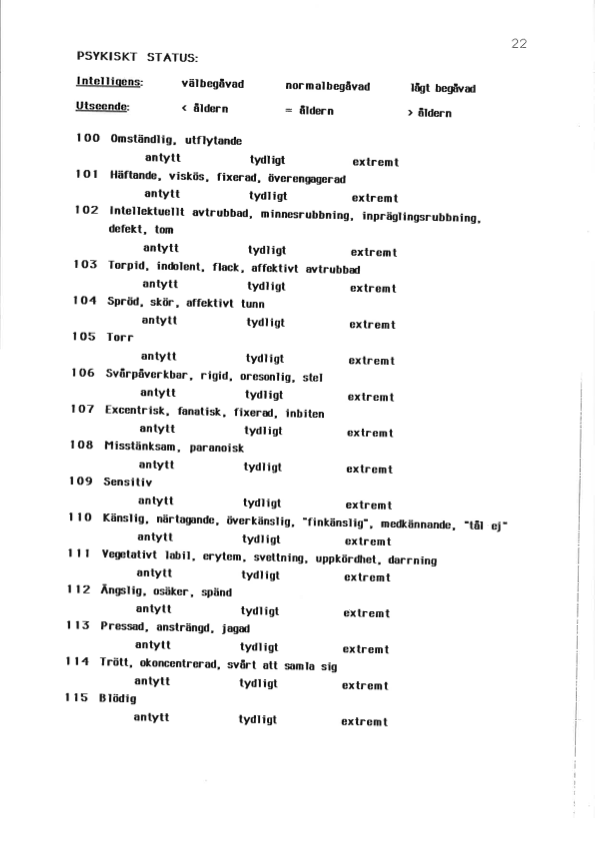

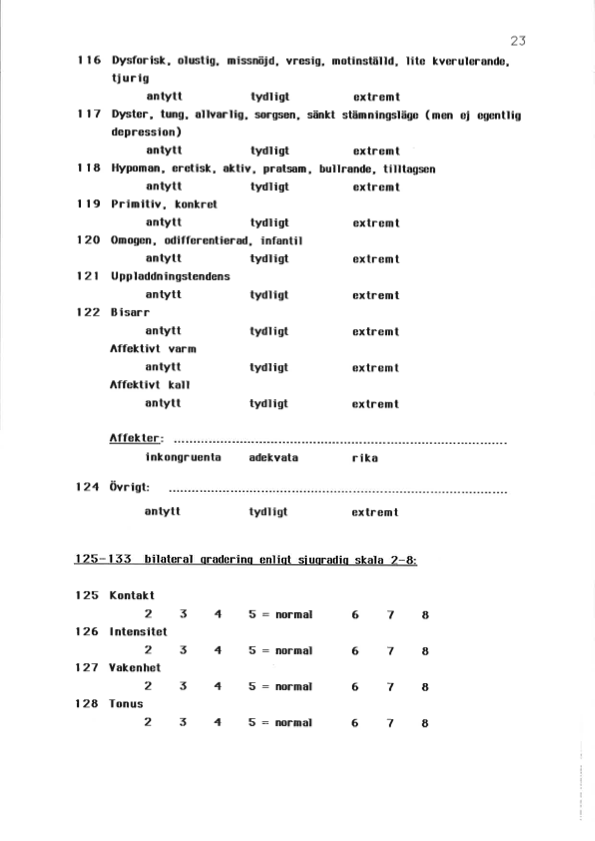

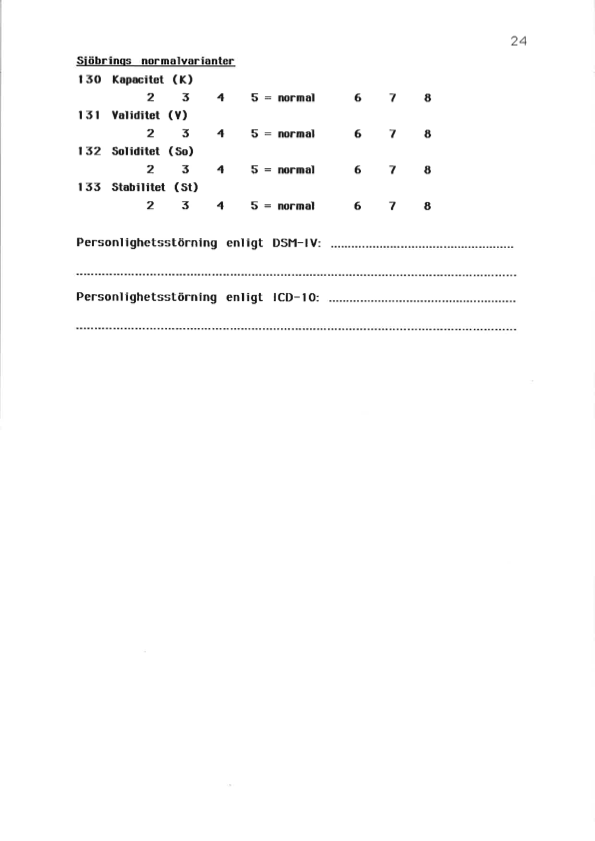

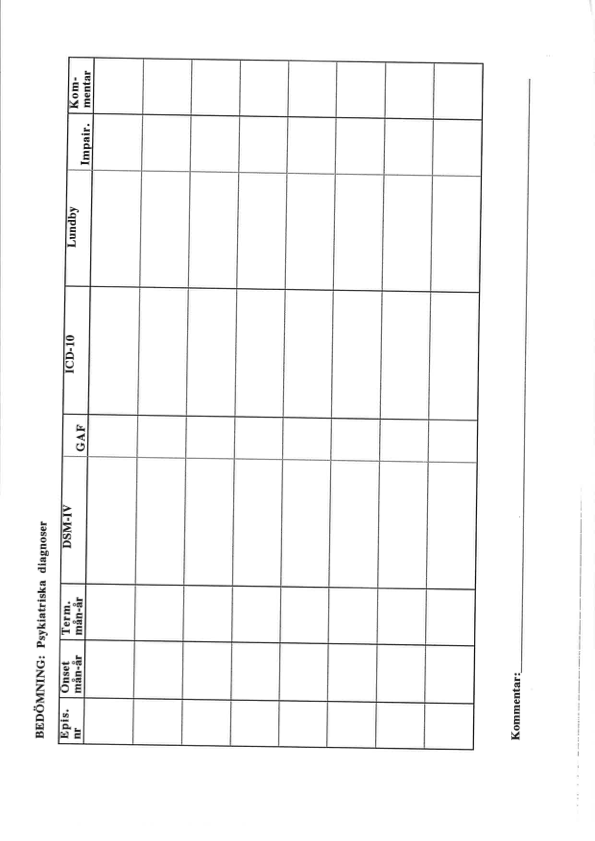


| Supplementary File 2  Overview of qualitative themes and subthemes and their quantitative proxies identified in the Lundby questionnaire. | |
| --- | --- |
| Qualitative themes | **Questions in the Lundby Study^[[1]](#footnote-1)^** |
| Mental health and well-being |  |
| Difficulties sleeping | How are you sleeping? (good/fairly good/poor)  *(Hur är det med sömnen? (bra/någorlunda/däligt))* |
| Feeling stressed | Have you experienced any severe difficulties or crisis since last follow-up? (no/yes)  *(Har du haft någon svår motgång/kris under perioden? (nej/ja))*  Have you had any mental health problems since last follow-up? (no/yes)  *(Har du haft psykiska besvär under perioden? (nej/ja))*  Are you restless? (often/sometimes/rarely/never)  *(Är du rastlös? (ofta/ibland/sällan/aldrig))*  Are you forgetgul? (often/sometimes/rarely/never)  *(Är du glömsk? (ofta/ibland/sällan/aldrig))* |
| Feeling anxious | Are you nervous? (often/sometimes/rarely/never)  *(Är du nervöst lagd? (ofta/ibland/sällan/aldrig))* |
| Feeling lonely | Do you feel lonely? (often/sometimes/rarely/never)  *(Känner du dig ensam? (ofta/ibland/sällan/aldrig))* |
| Suicidal thoughts | Suicidal thoughts/suicide attempt? (no/yes)  *(Suicidtanker/suicidforsök? (nej/ja))* |
| Feeling unhappy | How are you doing in life at the moment? (very good/good/fairly good/poor)  *(Hur trivs du med livet just nu? (utmärkt/bra/någorlunda/dåligt))* |
| Feeling tired/not having any energy | Do you feel that you are just as vital as those of the same age? (yes/no: more vital/less vital)  *(Tycker du att du är lika vital som dina jämnåriga? (ja/nej: mer vital mindre vital))*  Do you tire easily? (often/sometimes/rarely/never)  *(Blir du lätt trött? (ofta/ibland/sällan/aldrig))* |
| Cries a lot | Are you sensitive, do you cry easily? (often/sometimes/rarely/never)  *(Är du blödig, lätt for at gråta? (ofta/ibland/sällan/aldrig))* |
| Speculations | Do you easily feel shame? (often/sometimes/rarely/never)  *(Får du lätt skamkänslor? (ofta/ibland/sällan/aldrig))*  Do you care about what others think? (often/sometimes/rarely/never)  *(Bryr du dig om vad andra tanker och tycker? (ofta/ibland/sällan/aldrig))* |
| Physical health |  |
| Not feeling well | Do you feel well? (no/yes)  *(Känner du dig frisk? (no/yes))* |
| Having health problems | Current contact with the health care system? (no/yes). If yes, which area: primary care/psychiatry/other  *(Akutell sjukvårdskontakt? (no/yes), om ja: (primärvården/psykiatrin/annan))*  Gatric ulcer  *(Magsår)*  Stomach issues, other  *(Magbesvär, övriga)*  Surgical condition  *(Kirurgisk sjukdom)*  Orthopedic condition  *(Ortopedisk sjukdom)*  Back or joint issues  *(Rygg- el. ledbesvär)*  Heart or circulatory diseases  *(Hjärt/kärlsjukdom)*  Hypertension  *(Högt blodtryck)*  Chronic Obstructive Pulmonary Disease (COPD)  *(Kronisk obstruktiv lungsjuldom (KOL))*  Asthmatics  *(Astma)*  Other allergic diseases (hayfever, echsema)  *(Annan allergi (hösnuva, eksem))*  Dizziness  *(Yrsel)*  Headache, migraine or other  *(Huvedvärk, migrän, annan)*  Other kind of pain  *(Värk/smärta av annat slag)*  Had any scans  *(Skakningar)*  Encephalitis  *(Hjärnhinneinflammation)*  Brain scans  *(Hjärnskakning)*  Syncopes  *(Svimningar)*  Epilepsy  *(Epilepsi)*  Seizures of unknown kind  *(Anfall af uklar natur)*  Neurological disease  *(Neurologisk sjukdom)*  Memory problems  *(Minnesförlust)*  Kidney or urinary tract disease  *(Njursygdom/urinvägssjd)*  Skin disease  *(Hudsjukdom)*  Infections  *(Infektionssygdom)*  Hepatitis  *(Hepatit)*  Often common cold, easily infected  *(Ofta förkyld, infektionskänslig)*  Rheumatic fever  *(Reumatisk feber)*  Endocrinological disease  *(Endokrin sjukdom)*  Diabetes  *(Diabetes)*  Autoimmune disease  *(Autoimmunsjukdom)*  Cancer  *(Cancersjukdom)*  Eye or ear disease  *(Ögon/öronsjukdom)*  Obesity  *(Adipositas)*  Other disease  *(Annan sjukdom)*  Disability (minimally/very mild or mild/very/severe)  *(Handikapp (minimalt/mkt lätt el. lätt/medelsvårt/mkt svårt))* |
| The time before spousal death |  |
| Course of Illness before death | Have spouse had any physical illness? (no/yes)  *(Har make/maka haft någon kroppslig sjukdom? (nej/ja))*  Have spouse had any mental illness? (no/yes)  *(Har make/maka varit psykiskt sjuk? (nej/ja))*  Have spouse had any abuse of alcohol or pills? (no/yes)  *(Har make/maka missbrukat alkohol eller tabletter? (nej/ja))* |
| Practicalities |  |
| Shared responsibility of practicalities with spouse | How do(did) you consider the sharing of duties in your marriage? (very good/good/ordinary/less good/poor)  *(Hur upfattar/ade du arbetsgemenskapen i ert åaktenskap? (mkt god/god/ordinär/mindre god/dälig))* |
| Financial situation | The yearly net income of the family (more than enough/enough/not enough)  *(Familiens årsindkomst, netto (blir över/räcker/räcker ej))*  Financial contributions (no/yes)  *(Bidrag (nej/ja))*  Contact with social services? (no/yes)  *(Kontakt med socialförvaltningen? (nej/ja))* |
| Social relations |  |
| Children | How many children do you have? (0/1/2/3/4/5/6..)  *(Hur manga barn har du? (0/1/2/3/4/5/6..))* |
| Activities |  |
| Participates in activites | Hobbies? (a lot/some/few/none)  *(Fritidsintressen? (mange/några/få/inga))*  Are you active in any associations/clubs? (no/yes)  *(Är du aktiv i någon förening? (nej/ja))*  Do you experience your leisure time as meaningful? (very good/good/fairly good/poor)  *(Hur upplever du din fritid, meningsfull? (utmärkt/bra/någorlunda/däligt))*  Job(s) 1972-1997 (…),  Activity at labour market (full time/part time/retired)  *(Yrke/yrken 1972-1997 (…) Yrkesverksam: Heltid/deltid/pensionär))* |

1. Original questions from the questionnaire as asked in Swedish are written in italics. [↑](#footnote-ref-1)
